# Male hormones activate EphA2 to facilitate Kaposi’s sarcoma-associated herpesvirus infection: Implications for gender disparity in Kaposi’s sarcoma

**DOI:** 10.1371/journal.ppat.1006580

**Published:** 2017-09-28

**Authors:** Xing Wang, Zhe Zou, Zhaohui Deng, Deguang Liang, Xin Zhou, Rui Sun, Ke Lan

**Affiliations:** 1 State Key Laboratory of Virology, College of Life Science, Wuhan University, Wuhan, Hubei Province, China; 2 Hospital of Xinjiang Production and Construction Corps, Urumqi, Xinjiang, China; 3 Cancer Hospital of Xinjiang Medical University, Urumqi, Xinjiang, China; University of Southern California, UNITED STATES

## Abstract

There is increasing consensus that males are more vulnerable than females to infection by several pathogens. However, the underlying mechanism needs further investigation. Here, it was showed that knockdown of androgen receptor (AR) expression or pre-treatment with 5α-dihydrotestosterone, the AR agonist, led to a considerably dysregulated Kaposi’s sarcoma-associated herpesvirus (KSHV) infection. In endothelial cells, membrane-localized AR promoted the endocytosis and nuclear trafficking of KSHV. The AR interacted with ephrin receptor A2 (EphA2) and increased its phosphorylation at residue Ser897, which was specifically upregulated upon KSHV infection. This phosphorylation resulted from the AR-mediated recruitment of Src, which resulted in the activation of p90 ribosomal S6 kinase 1 (RSK1), which directly phosphorylates EphA2 at Ser897. Finally, the EphA2-mediated entry of KSHV was abolished in a Ser897Asn EphA2 mutant. Taken together, membrane-localized AR was identified as a KSHV entry factor that cooperatively activates Src/RSK1/EphA2 signaling, which subsequently promotes KSHV infection of both endothelial and epithelial cells.

## Author summary

Although KS incidence is higher in males, which correlates with higher seroprevalence and viral DNA levels in the blood, little is known whether male sex steroids contribute to this disparity. In the present study, we have confirmed the role of both AR and its ligand in promoting KSHV primary infection in target cells. Specifically, AR inhibition led to a dramatically decreased number of perinuclear-accumulated virus particles during early KSHV entry stage. Mechanically speaking, the effect was resulted from the interaction of AR with known KSHV receptor EphA2 and stimulating signal transduction. The AR recruited Src, activated RSK1, and then increased EphA2 phosphorylation at residue Ser897, which is prerequisite for successful KSHV infection. Our study provides for the first time a unique insight into why KSHV may have a higher prevalence in males.

## Introduction

Males of many species are more susceptible than females to infections caused by parasites, fungi, bacteria, and viruses. Among humans, there is a reported male predominance in the prevalence and lethality of infections with various pathogens. This may reflect different exposures and immune responses, or even differences in genetic susceptibility between genders [1–3]. Sex-based differences typically become apparent after puberty, which suggests a role of steroid hormones in pathogenesis. Most current studies have investigated this discrepancy in terms of gender-specific immune responses, and the results showed that females have a greater ability to produce immune responses against infections. 17β-Estradiol regulates the activity of immune cells, including lymphocytes, macrophages, granulocytes, and mast cells [4, 5]. A lack of the inhibitory factor CD200R in females leads to Toll-like receptor 7-mediated activation of interferon-α, which accounts for higher immune status in females, at least in a murine model [6–8].

Additionally, sex hormones can directly affect pathogen infections. Higher serum androgen levels and an androgen receptor (AR) gene containing shorter CAG repeats (which lead to higher AR activity) have been clinically linked to higher risks of hepatitis B virus (HBV)-mediated hepatocellular carcinoma (HCC) [9]. The AR increases HBV genome replication by binding to two androgen-responsive elements that are located in enhancers I and II of HBV, which strongly implicates male gender as a risk factor for HCC development [10, 11]. Correspondingly, estrogen and the estrogen receptor repress the transcription of HBV genes by binding competitively with hepatic nuclear factor 4α to enhancer I [12]. However, whether male sex hormones function in the pathogenesis of other human viruses remains largely unknown.

Kaposi’s sarcoma (KS), at least the classical and endemic types, occurs disproportionately in men [13–18]. The age-standardized incidence rate of KS was 12.3 and 4.6 per 100,000 in African males and females, respectively. In older age groups, KS was about 10 times more common in males [19]. Regarding the gender-associated seroprevalence of KS-associated herpesvirus (KSHV), the causative agent of KS, a recent evidence-based meta-analysis indicated that KSHV preferentially infects males in Africa [20], and a significantly higher quantity of KSHV DNA has been detected in men than women [21, 22]. These data strongly suggest that male hormones may play a role in KSHV infection and pathogenesis. However, whether and how the hormone system is involved in these processes still remains unknown.

The classical role of the AR is that of a steroid hormone-activated transcription factor. Intracellular AR translocates into the nucleus and then stimulates the transcription of androgen responsive genes after binding its hormone ligand. However, another category of membrane-localized AR in Lipid Rafts (LRs) was identified a decade ago, and its biological significance remains unknown [23, 24]. Here, we demonstrate that membrane-localized AR can promote KSHV infectivity, especially at the early entry stage. Both AR and 5α-dihydrotestosterone (DHT), the agonist of AR, promoted KSHV infection, as determined by a quantitative real-time polymerase chain reaction (qRT-PCR) assessment of the copy number of the KSHV genome and its transcripts. This effect was mediated by association with, and the consequent phosphorylation of ephrin receptor A2 (EphA2), one of the major KSHV entry receptors [25, 26]. The specific residue Ser897 of EphA2 was identified as an essential phosphorylation site responsible for KSHV entry. Interestingly, the Ser897 phosphorylation of EphA2 can be induced by the AR-mediated recruitment of Src, which led to the activation of the kinase p90 ribosomal S6 kinase 1 (RSK1), which directly phosphorylates EphA2. These findings demonstrated that male sex hormones facilitate KSHV primary infection through a Src/RSK1/EphA2 Ser897 signaling cascade and may imply a novel mechanism for gender disparity in KS incidence.

## Results

### AR facilitates KSHV primary infection of both endothelial cells and epithelial cells

**Fig 1:**
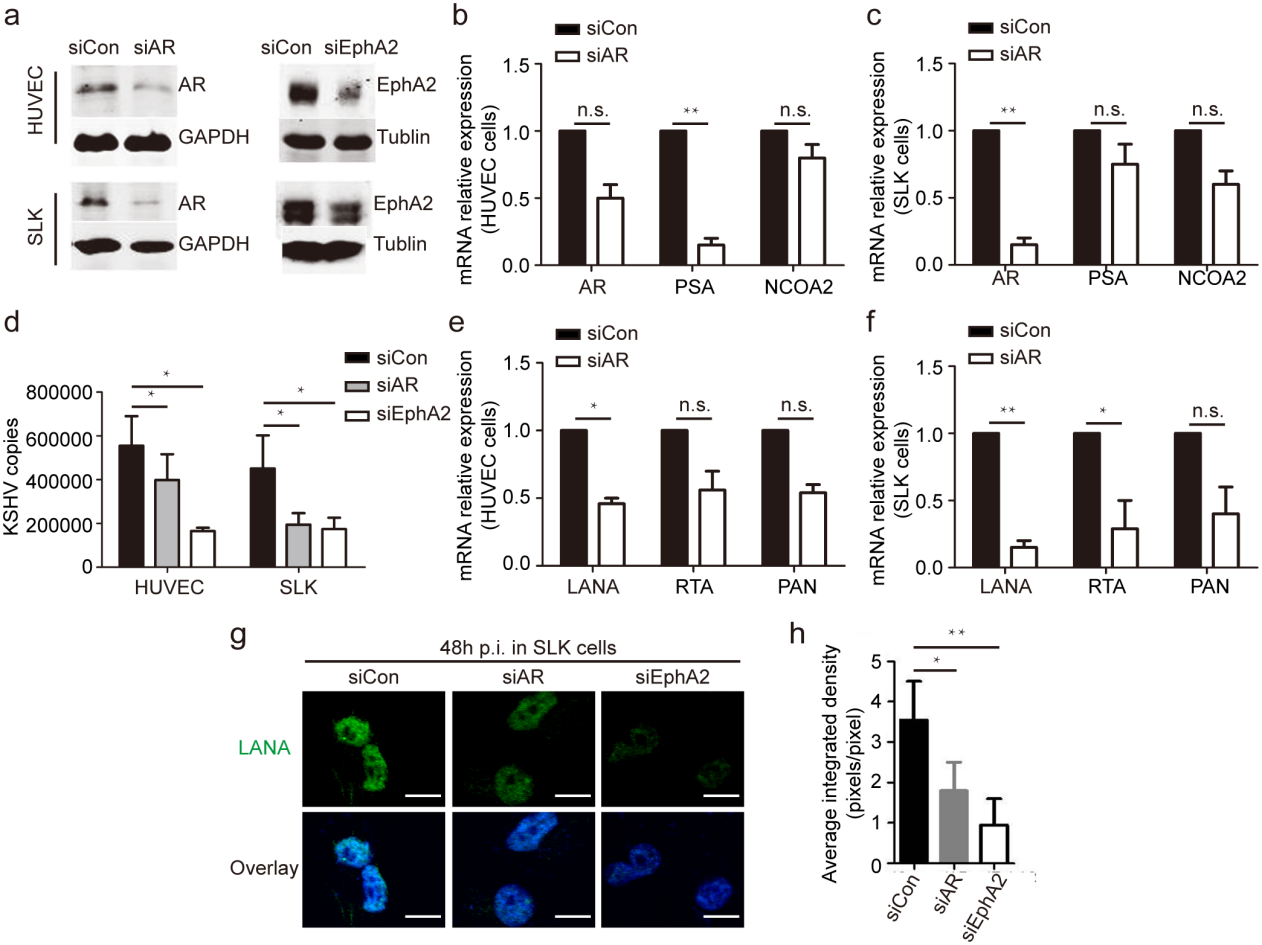
Knockdown of AR expression decreases the efficiency of KSHV infection in endothelial cells. (a) Endogenous AR expression in HUVECs and SLK cells is efficiently impaired by RNA interference of the AR. siRNAs were transfected into cells at final concentrations of 100 nM for HUVECs and 200 nM for SLK cells using Lipofectamine 2000. Cell lysates were subjected to immunoblotting using the indicated antibodies. (b, c). RNA transcription of the AR and its target genes is inhibited by AR siRNA treatment in both HUVECs (b) and SLK cells (c). Total RNA from the above samples was subjected to a qRT-PCR to determine the mRNA levels of the indicated genes. Values were normalized to the level of actin mRNA and calibrated by comparison to a control siRNA treatment. (d) AR inhibition leads to a significant reduction of the number of internalized KSHV DNA copies. The above cells were infected with KSHV for 2 h at 37°C, washed, and cultured for another 22 h, and the MOIs were 10 and 5 for HUVECs and SLK cells, respectively. Cellular genomic DNA was extracted, and internalized KSHV DNA was quantitated by amplification of the LANA gene by qRT-PCR. (e, f) The mRNA expression of KSHV genes is down-regulated by AR inhibition. Total RNA from the above HUVECs (e) and SLK cells (f) was subjected to qRT-PCR to determine the expression of LANA, RTA, and PAN. The data were normalized to the actin expression and then calibrated by the control siRNA treatment. (g, h) Knockdown of AR and EphA2 expression decreases nuclear LANA expression in SLK cells, as determined by an immunofluorescence assay and quantitative analysis by Image J software. The infection time was 48 h in this group. Scale bars represent 10 μm. The randomly selected fifty cells in each image were analyzed and the corrected total cell fluorescence (CTCF) of individual images was calculated as following: CTCF = Integrated Density–(Area of selected cells X Mean fluorescence of background readings). Representative images are shown. Data are shown as the mean±SEM; n = 3. Paired Student’s *t*-tests and one-way ANOVA analysis was respectively performed on (b, c, e and f) and (d, h). * p,0.05, ** p,0.01, ^n.s.^ p, no significance.

As the common function of LRs in promoting KSHV primary infection [27], we speculated that co-localized AR may play a concordant role in KSHV infection of target cells. KSHV had a broad tropism in vivo in a variety of cell types such as endothelial cells, epithelial cells, monocytes and keratinocytes. Herein, primary human umbilical vein endothelial cells (HUVECs) and a culture of epithelial-cell origin (SLK cells) were employed to analyze the role of the AR in KSHV infections through RNA interference; a small interfering RNA (siRNA) targeting EphA2 was used as a positive control since EphA2 is known to be the entry receptor of KSHV infection for these cells [25, 26]. The inhibitory effect of the AR siRNA was demonstrated by reduced AR expression (Fig 1a–1c) and the consequent inability to upregulate the transcription of AR target genes, the prostate-specific antigen (PSA) and nuclear receptor coactivator 2 (NCOA2) genes [28] (Fig 1b and 1c). The specificity of AR detection was confirmed by its abundance of 110 kDa full-length isoform and the typical nuclear localization in androgen-sensitive cells (S1 Fig).

**Fig 2:**
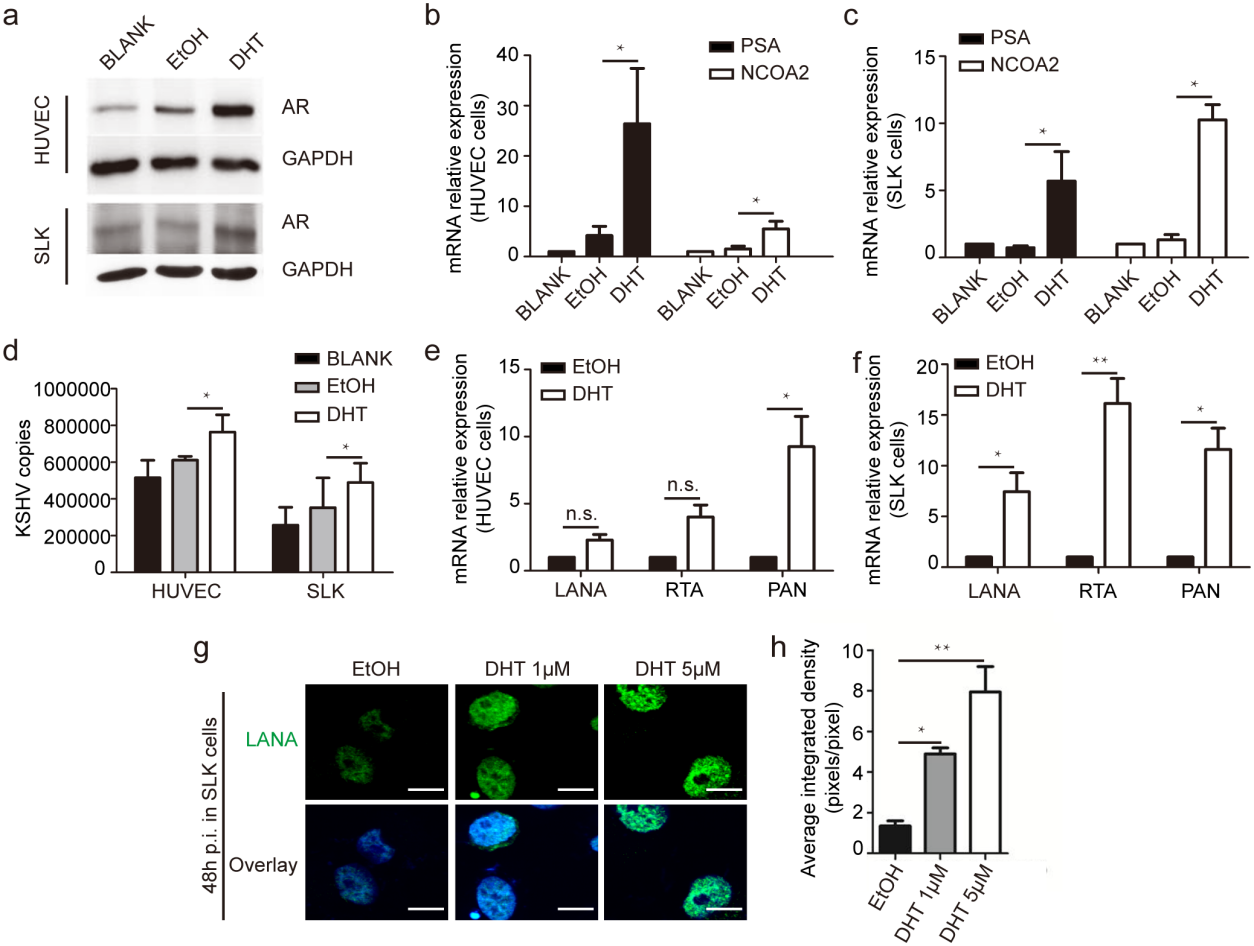
Androgen, the ligand of AR, also facilitates KSHV primary infection in HUVECs and SLK cells. (a) DHT treatment increases AR expression in HUVECs and SLK cells. HUVECs and SLK cells were left untreated or treated by DHT at 1 uM or 3 uM respectively, or its solvent, ethanol at 0.1–0.3% (v/v), for 24 h and were subjected to immunoblotting with the indicated antibodies. Charcoal-stripped FBS was used for cell culture. (b, c) The expression of AR target genes increases in response to DHT treatment in HUVECs (b) and SLK cells (c). Cellular RNA was prepared from the same samples, and the mRNA expression of the AR, PSA, and NCOA2 genes was determined by normalization to actin gene expression and then compared with untreated cells. (d, e, f) DHT treatment increases the KSHV genome copy number and the transcription of viral genes. Cells were treated as described above and infected for 24 h with KSHV at an MOI of 10 for HUVECs and 5 for SLK cells. The extraction of total DNA and RNA and the following analysis were performed as previously described and the normalization control of GAPDH was utilized. (g, h) DHT treatment considerably increases LANA-positive nuclear staining in SLK cells. The SLK cells were infected by KSHV for 48 h and subjected to immunofluorescence detection and fluorescent density was quantified by Image J. Scale bars represent 10 μm. The fifty cells in each images were selected and the CTCF of individual images were calculated as mentioned in Fig. 2. Data are shown as the mean±SEM; n = 3. Paired Student’s t-tests and one-way ANOVA analysis was performed on (b, c e, f) and (d, h) respectively. * p,0.05, ** p,0.01, ^n.s.^ p, no significance.

Furthermore, AR inhibition led to a dramatic reduction of KSHV infection, as determined by measuring the number of internalized KSHV copies of the LANA gene in HUVECs and SLK cells (Fig 1d). Only 398,000 viral copies were internalized in AR siRNA-treated HUVECs, compared with 555,000 KSHV copies in control siRNA-treated HUVECs (a 28% reduction), while those for SLK cells were 194,000 and 451,000, respectively (a 57% reduction) (Fig 1d). Expectedly, the mRNA levels of viral genes in HUVECs with AR knockdown was considerably decreased, as indicated by 54, 44, and 46% reductions in the transcription of the LANA, replication and transcription activator (RTA), and polyadenylated nuclear RNA (PAN) genes, respectively (Fig 1e), and by 85, 71, and 60% reductions, respectively, in SLK cells (Fig 1f). Accordingly, compared with the control groups, at 48 h post-infection (p.i.), we observed a dramatic inhibition of nuclear LANA immunostaining in AR and EphA2 siRNA-treated SLK cells (Fig 1g and 1h). Importantly, we found that not only the AR, but also its ligand, DHT, were capable of increasing KSHV infection in HUVECs and SLK cells (Fig 2). This was also validated in lymphatic endothelial cells (LECs), another well-established endothelial cell model for in vitro KSHV infection (S2 Fig). Collectively, these results strongly suggest that both the AR and its ligand are able to facilitate KSHV infection in various cell types.

### The AR is required for productive endocytosis and trafficking of KSHV

KSHV infection of endothelial cells consists of multiple steps [29, 30], therefore it is necessary to define the stage at which AR facilitated KSHV infection. As lipid rafts (LRs), where AR is located, have been shown to be essential for the post-binding and entry stages of KSHV infection [27, 31], we hypothesized that the AR may also contribute to this process. It was reported that KSHV enters the cells through endocytosis and it should retain its envelope immediately after internalization but lose it when subsequent fusion with endosomal membrane occurs, thus the glycoprotein B (gB) is one of the viral markers to indicate the early stage of KSHV endosome trafficking [32]. Here, the intracellular gB staining was used to represent early stage of KSHV entry and trafficking.

**Fig 3:**
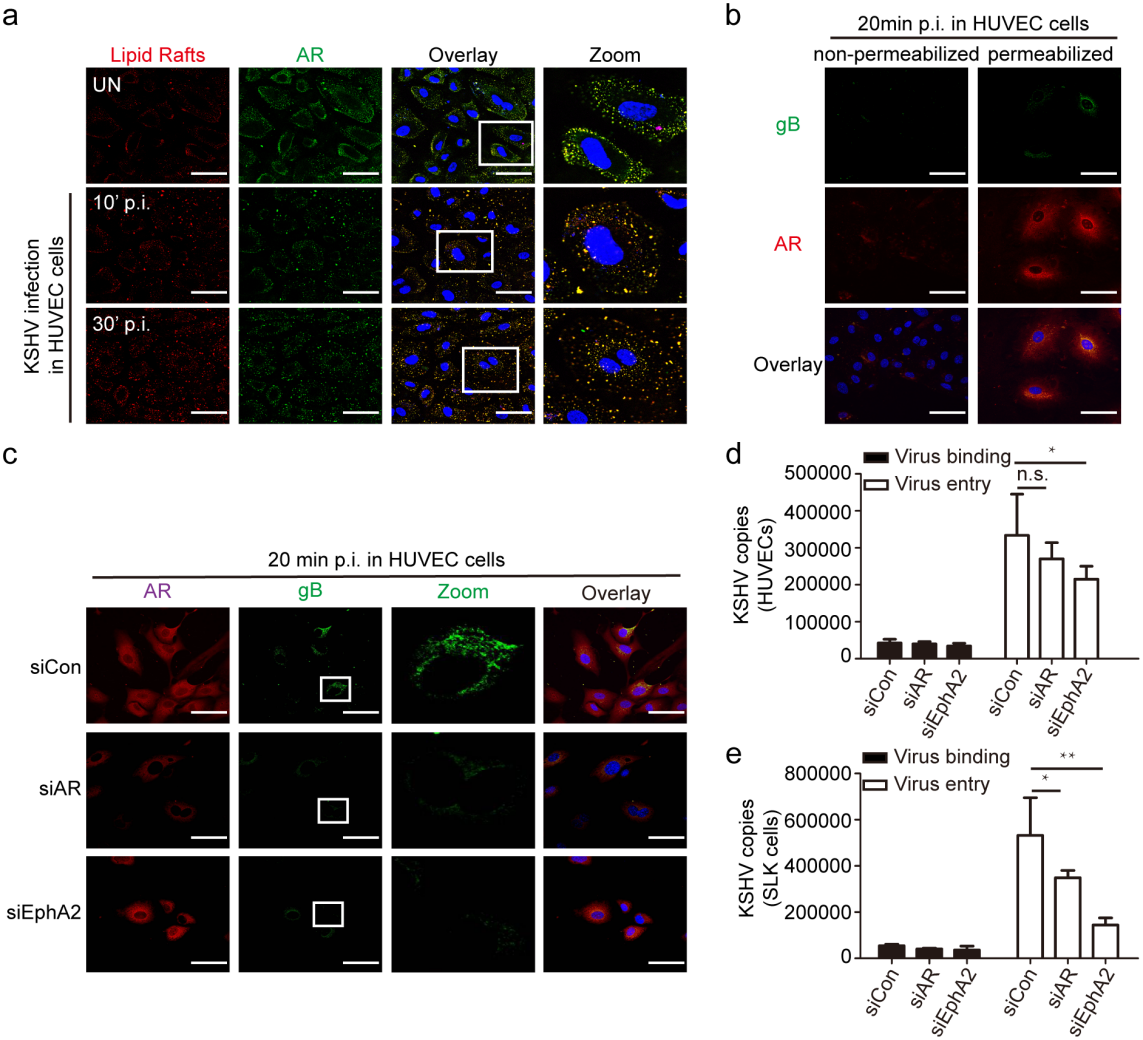
Membrane-localized AR facilitates productive endocytosis and nuclear trafficking of KSHV. (a) Upon KSHV infection, membrane-localized AR translocates into the nucleus. HUVECs were left uninfected or infected by KSHV (MOI = 10) for the indicated times. After washing, the cells were processed for immunofluorescence of α-CtB (an LR marker, red), and then fixed and stained for α-AR (green) and nuclei (blue). Scale bars represent 40 μm. Insets show the enlarged images of the boxed areas. (b) Accumulated perinuclear expression of gB was observed exclusively in permeabilized cells at 20 minutes p.i. Cells were performed as mentioned above, except lacking of Triton X-100 treatment in non-permeabilized group. Scale bars represent 50 μm. (c) Knockdown of AR and EphA2 expression reduces KSHV endocytosis, as indicated by decreasing gB staining in cells. HUVECs were transfected by siRNAs at 100 nM for 6 h and then cultured in fresh medium for another 18 h. At 20 min p.i. at an MOI of 10, the cells were reacted with α-gB (green), α-AR (red), and DAPI (blue). Scale bars represent 50 μm. Representative images are shown. Each reaction was repeated in, at least, triplicate. (d, e) The suppressive effect of AR and EphA2 inhibition on KSHV entry, but not binding, was determined by measuring the numbers of internalized KSHV viral DNA copies in HUVECs (d) and SLK cells (e). siRNA-transfected cells were infected by KSHV for 1 h at 4 or 37°C. After washing, total DNA was isolated and subjected to real-time DNA PCR of the LANA gene as described previously. For virus entry detection, an extra 0.25% trypsin-EDTA treatment for 5 min at 37°C, after washing with PBS, was used to remove bound, but not internalized, viruses. Data are shown as the mean±SEM; n = 3. One-way ANOVA analysis was performed on (d) to (e). * p,0.05, ** p,0.01, ^n.s.^ p, no significance.

As shown in Fig 3a, the localization of membrane-localized AR in LRs was confirmed in HUVECs. We further observed that the translocation of the AR from the membrane and cytoplasm into the nucleus occurred immediately upon KSHV infection, as early as 30 min p.i. (Fig 3a). In Fig 3b, the successful internalization and perinuclear accumulation of gB-positive KSHV particles were observed only in permeabilized cells, accompanied by AR nuclear translocation. The specificity of fluorescent gB and AR expression at KSHV early entry stage was verified by involving mock staining for the two molecules, which precisely exclude the contaminant green or red fluorescent signals from rKSHV.219 virus (S3 Fig). The specificity of fluorescent labeling of LRs was verified by concordant pattern of LR localization between B cells [33] (S4a Fig). And the co-localization between gB and early endosome marker EEA1 (Early Endosome Antigen 1) was identified at 20’ p.i. in HUVEC cells indicating the successful KSHV early endocytosis (S4b Fig). We next examined the efficiency of KSHV endocytosis upon AR siRNA treatment. The results demonstrated a dramatic reduction of the internalized perinuclear staining of gB (green) in AR siRNA-treated cells (Fig 3c). As a positive control, treatment with EphA2 siRNA had a greater effect on KSHV internalization and accumulation (Fig 3c). On the contrary, DHT treatment increased the number of KSHV virions that reached the nuclear periphery region in HUVECs (S4e Fig) and LECs (S4f Fig) as well. Next, we assessed the role of the AR in KSHV binding and entry. Compared with the control siRNA, the AR siRNA inhibited KSHV entry, as determined by significant reductions in the number of internalized KSHV DNA copies in HUVECs (by 19.2%) and SLK cells (by 36%), but it did not affect KSHV binding (Fig 3d and 3e). In contrast to that, being control virus of Herpes Simplex Virus 1 (HSV1) which independent of EphA2 as cellular receptor [34], inhibition of either AR or EphA2 had no effect on virus binding and entry (S4c and S4d Fig). Collectively, these results demonstrated that membrane-localized AR can facilitate KSHV infection in the early entry stage, rather than the binding stage.

### AR and its ligand promote the phosphorylation of EphA2 at Ser897, which is essential for KSHV entry

**Fig 4:**
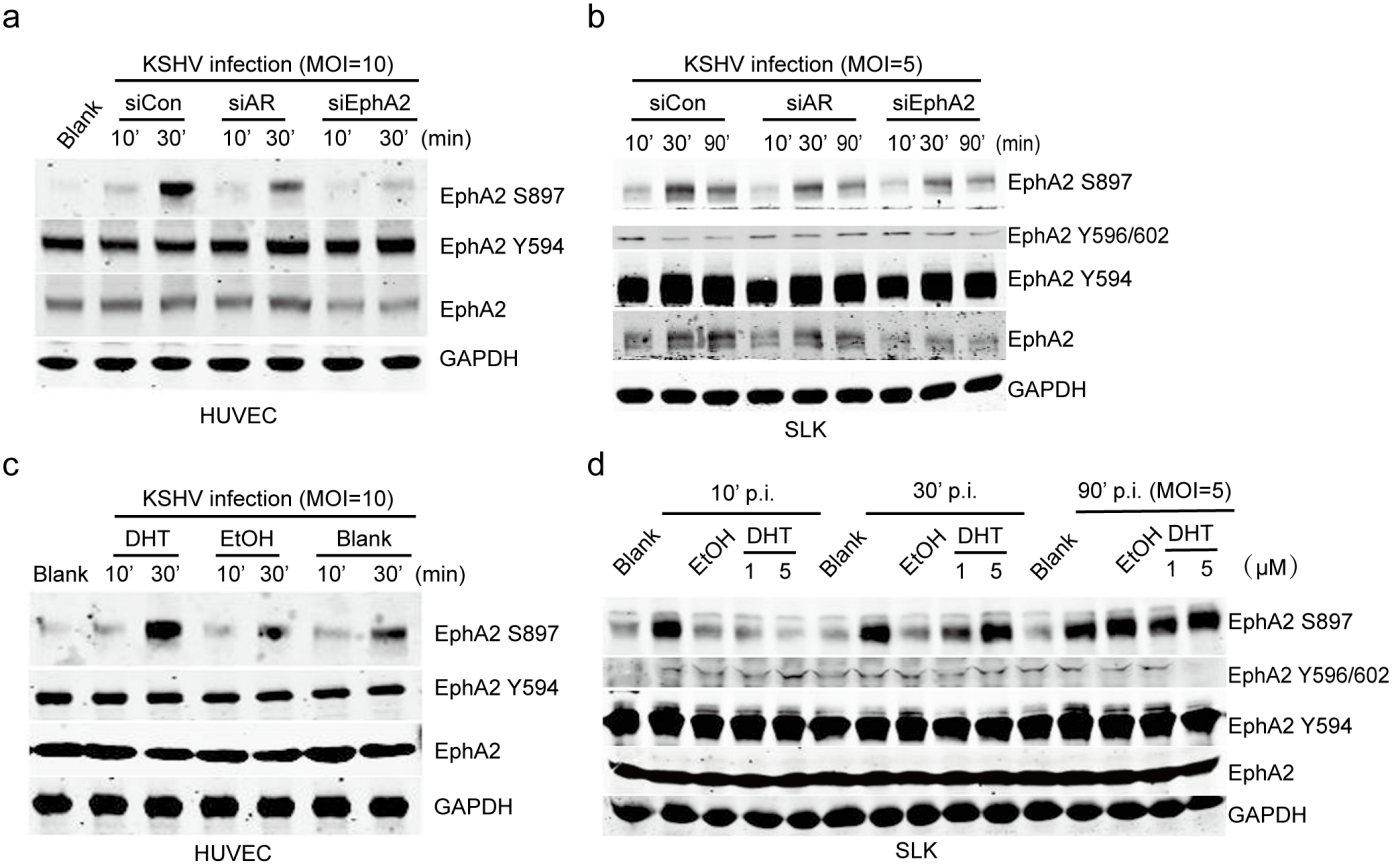
AR and its ligand specifically promote the phosphorylation of EphA2 at Ser897. (a, b) The level of EphA2 that is phosphorylated at Ser897 is specifically upregulated upon KSHV infection, and the effect is significantly inhibited by AR and EphA2 siRNA treatment. siRNAs were transfected into cells at final concentrations of 100 nM for HUVECs and 200 nM for SLK cells using Lipofectamine 2000. siRNA-transfected cells were serum-starved for 6 h prior to the inoculation of KSHV at the indicated MOIs for the indicated times. Cells were lysed in the presence of protease and phosphatase inhibitors and subjected to western blotting. (c, d) DHT treatment specifically increases EphA2 phosphorylation at Ser897 upon KSHV infection. Cells were left untreated, or treated with DHT or ethanol at 1 or 3 uM as described previously, infected with KSHV, and lysed as described above. Each reaction was repeated in, at least, triplicate.

As shown above, the AR participated in KSHV endocytosis. Being a member of the largest family of tyrosine kinase receptors, EphA2 has been defined as a KSHV receptor that is required for virus entry, at least in adherent cells [25, 26]. It was previously demonstrated that the intracellular kinase domain of the EphA2 receptor is indispensable for KSHV entry [25], thus we hypothesized the role of AR in mediating the catalysis of EphA2. Because the specific phosphorylation sites that account for EphA2 phosphorylation have not been reported, we first attempted to identify the sites activated by KSHV infection. The results showed that the phosphorylation of EphA2 at Ser897, but not that of other tyrosine phosphorylation sites, e.g., Y594 or Y596/602, is specifically upregulated by KSHV infection in both HUVECs and SLK cells (Fig 4). EphA2 was rapidly phosphorylated at Ser897 at 10 min p.i., and the phosphorylation significantly increased and persisted for 30 min p.i. in HUVEC, whereas a reduction by 90 min p.i. was observed in SLK cells (Fig 4a and 4b). In addition, the AR siRNA had a suppressive effect on EphA2 Ser897 phosphorylation (Fig 4a and 4b).

Consistent with this, DHT treatment led to a significant enhancement of the phosphorylation of EphA2 at Ser897 upon KSHV infection (Fig 4c and 4d). Additionally, DHT alone induced EphA2 Ser897 phosphorylation in SLK cells in the absence of KSHV infection (S5c Fig). At 30 and 90 min p.i., DHT increased the level of phosphorylated EphA2 Ser897 in a dose-dependent manner (Fig 4d). Additionally, DHT treatment further promoted the nuclear translocation of EphA2 that was phosphorylated at Ser897 (S5a Fig), and the effect was synergistically promoted by KSHV infection (S5b Fig).

**Fig 5:**
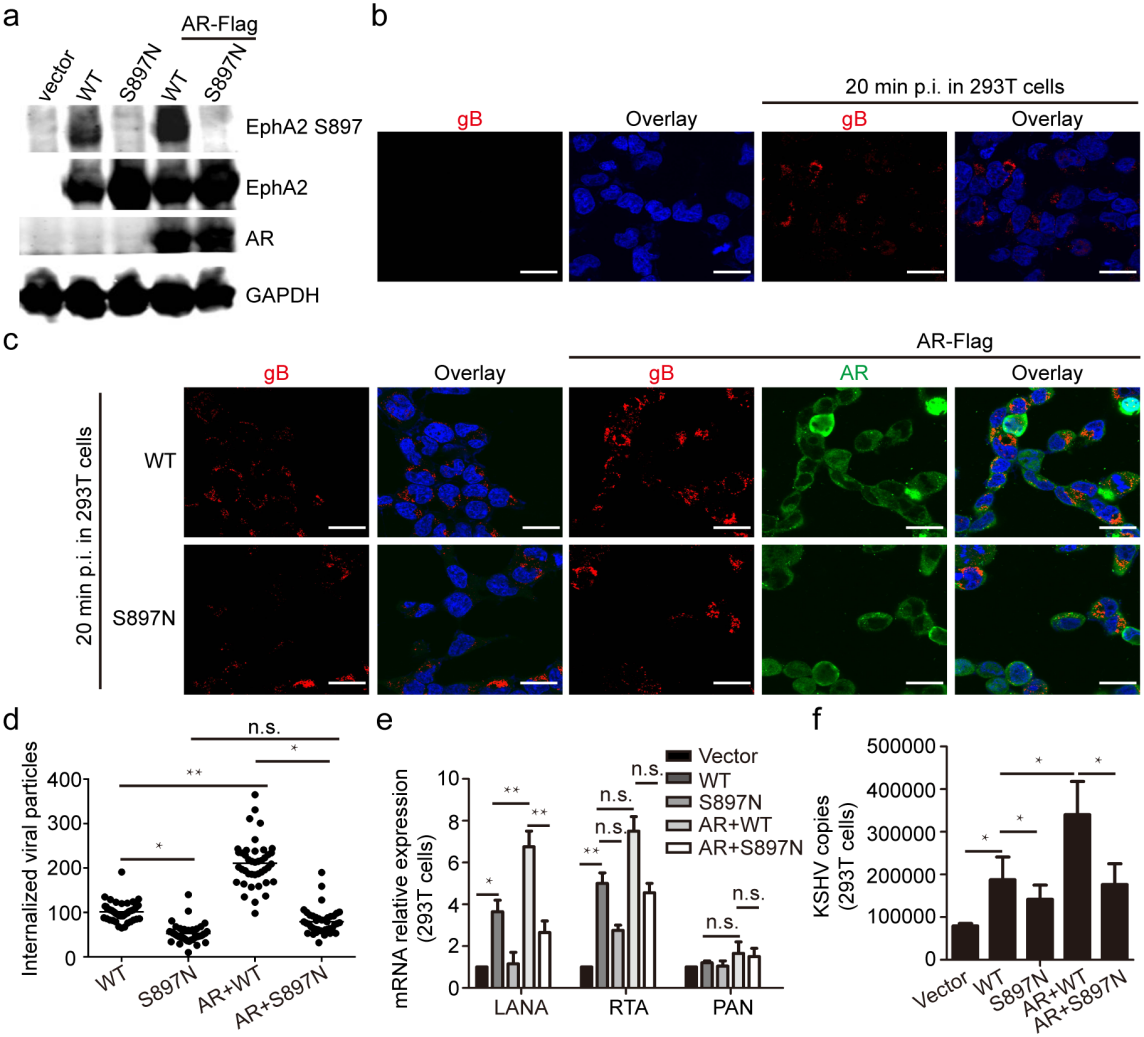
EphA2 phosphorylation at Ser897 is critical for AR-mediated KSHV entry. (a) The Ser897Asn mutation abolishes the phosphorylation of EphA2 by the AR. Plasmids expressing WT EphA2 or its site-specific mutant (S897N) were transfected into 293T cells, with or without co-transfection with a plasmid expressing FLAG-tagged AR. (b, c) Increased nuclear accumulation of KSHV virions by ectopic WT EphA2 is abolished by the S897N mutant and is not rescued by co-transfection with a plasmid expressing FLAG-tagged AR. 293T cells in (a) were infected with KSHV infection (MOI = 1) for 20 min. Cells were processed for immunofluorescence using the indicated antibodies. Each reaction was repeated in, at least, triplicate. Scale bars represent 40 μm. Representative images are shown. (d) The internalized viral particles represented by RFP signal intensity in cells from (c) were analyzed and counted by using Image pro-primier software. (e, f) The EphA2 S897N mutant greatly impairs expression of KSHV viral genes and internalized virions compared with WT EphA2. Cellular RNA and genomic DNA in (b and c) were extracted, respectively, and were subjected to qRT-PCR detection of the expression of LANA, RTA and PAN, or were quantitated by amplification of the LANA gene by qRT-PCR. Data are shown as the mean±SEM; n = 3. One-way ANOVA analysis and unpaired Student’s *t*-tests were performed on (e) and (d, f) respectively. * p,0.05, ** p,0.01, ^n.s.^ p, no significance.

To define the role of EphA2 Ser897 phosphorylation in KSHV entry, we constructed a mutant, named EphA2 Ser897Asn, in which Ser897 was mutated to Asn. This mutation completely abolished the phosphorylation of Ser897 of wild type EphA2, without affecting the total level of EphA2 (Fig 5a). In addition, the capability of ectopic AR to increase the level of phosphorylated EphA2 was nearly eliminated by the mutant (Fig 5a). Upon KSHV infection, we observed a large amount of KSHV virions in cells that were transfected with a plasmid expressing wild-type (WT) EphA2; however, this effect was eliminated in cells that were transfected with a construct expressing the EphA2 Asn897 mutant (Fig 5b and 5c). The results were verified by quantitative analysis to the internalized viral particles represented by red signals (Fig 5d and S6a Fig). Finally, we demonstrated that ectopic AR-induced internalization of KSHV virions is dramatically inhibited by the Ser897Asn mutant, and the results were verified by quantitative analysis showing decreased viral gene expression and KSHV copies (Fig 5c, 5e and 5f). Taken together, these data demonstrate that phosphorylation at Ser897 of EphA2 has a primary role in KSHV entry, and the consistent modulation of Ser897 phosphorylation by the AR and its ligand suggest that it is one of the possible mechanisms by which male hormones facilitate KSHV infection.

### AR forms a complex with activated EphA2 and Src during KSHV early infection

**Fig 6:**
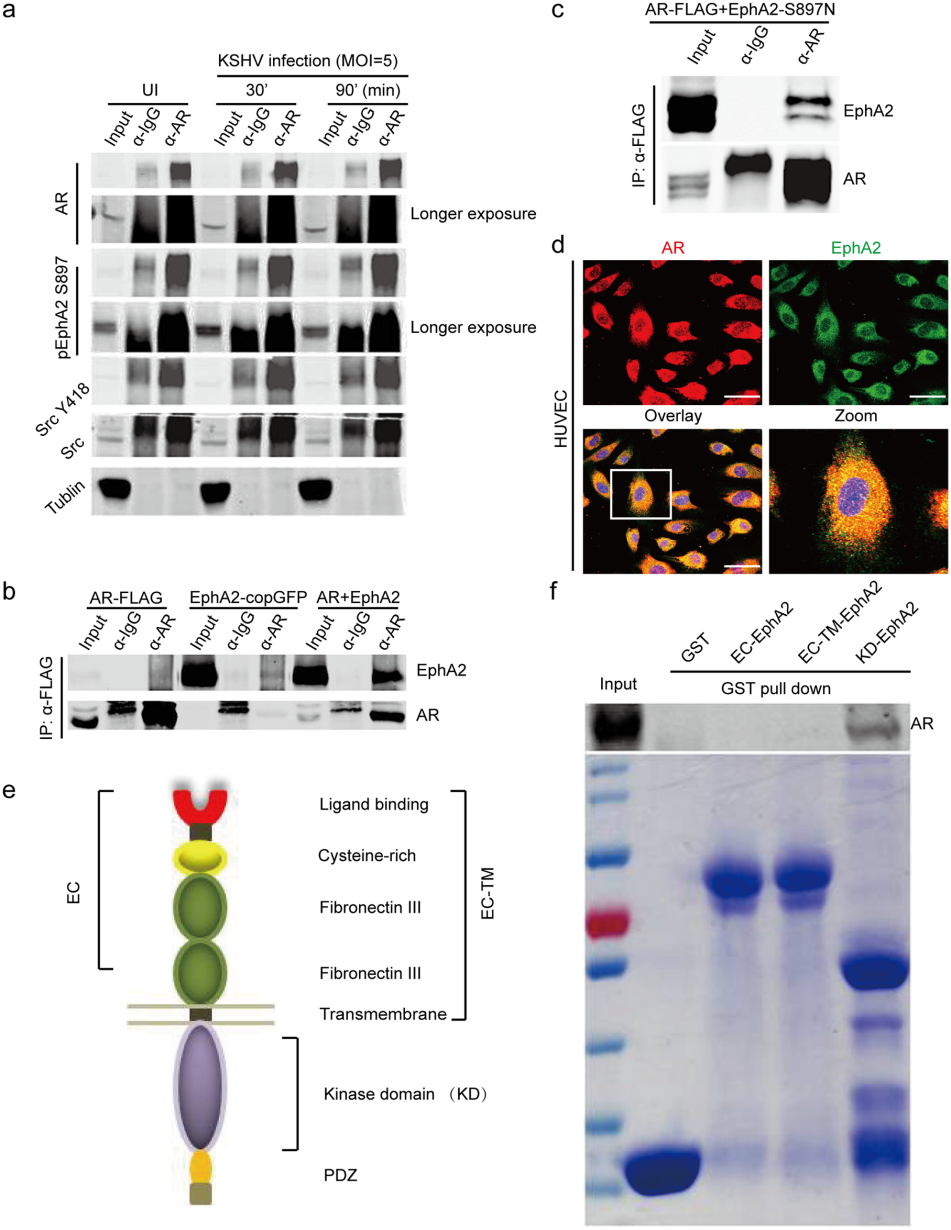
The AR interacts with the KSHV receptor EphA2 during the early infection stage. (a) The AR co-immunoprecipitates with the KSHV entry receptor EphA2 during KSHV infection. SLK cells were serum-starved for 6 h prior to KSHV infection. At the indicated times, uninfected or KSHV-infected cells were immunoprecipitated by an α-AR antibody and analyzed by western blotting. (b, c) The interaction between AR with wild-type EphA2 and AR with EphA2 Ser897Asn mutant were confirmed by the absence of KSHV infection. Plasmids expressing FLAG-tagged AR, wild-type EphA2 or EphA2 Ser897Asn mutant were transfected into 293T cells individually or in indicated combination.48 h later, the cells were subjected to immunoprecipitation using anti-FLAG M2 affinity beads, followed by western blotting. (d) The AR co-localizes with EphA2 in HUVECs. Cells were reacted with α-AR (red) and EphA2 (green) antibodies. Scale bars represent 40 μm. Representative images are shown. (e, f) The AR associates with the intracellular kinase domain of EphA2. Three GST-fused truncations of EphA2 were constructed: EC-EphA2 (the extracellular domain), EC-TM EphA2 (the extracellular domain plus transmembrane domain), and KD-EphA2 (the kinase domain) (e). GST affinity beads were pre-loaded with the GST-fused proteins or GST, and then incubated with in vitro-translated AR. Each reaction was repeated in, at least, triplicate.

To explore the mechanism by which AR activates EphA2, we first attempted to determine whether AR functions by directly binding to EphA2. As shown in Fig 6a, weconfirmed that AR can co-immunoprecipitates with activated EphA2 during KSHV primary infection, along with Src. The co-localization of the AR with Src was also verified [35] (S6b Fig). Importantly, we observed that the AR associated with EphA2 that is phosphorylated at Ser897 in KSHV-infected SLK cells, which was maximized at 90 min p.i. (Fig 6a). In addition, ectopic EphA2 was efficiently immunoprecipitated by an α-FLAG antibody, which recognizes FLAG-tagged AR, when these proteins were co-expressed in human embryonic kidney 293T cells and the interaction was remained between AR and EphA2 Ser897Asn mutant (Fig 6b and 6c). Moreover, confocal microscopy revealed that the AR co-localized with EphA2 on the cell membrane of HUVECs, as well as in the cytoplasm (Fig 6d). To map the exact EphA2 domains that are responsible for these associations, three glutathione S-transferase (GST) fused truncations of EphA2 were accordingly constructed [36] (Fig 6e). Finally, in vitro translated AR specifically bound to the kinase domain of EphA2, as determined by a GST pulldown assay (Fig 6f). Taken together, these studies suggest that the AR may promote KSHV endocytosis as a host entry factor by interacting with EphA2 and host signaling molecules.

### AR activates RSK1, the kinase that directly phosphorylates EphA2 at Ser897, by recruiting Src

**Fig 7:**
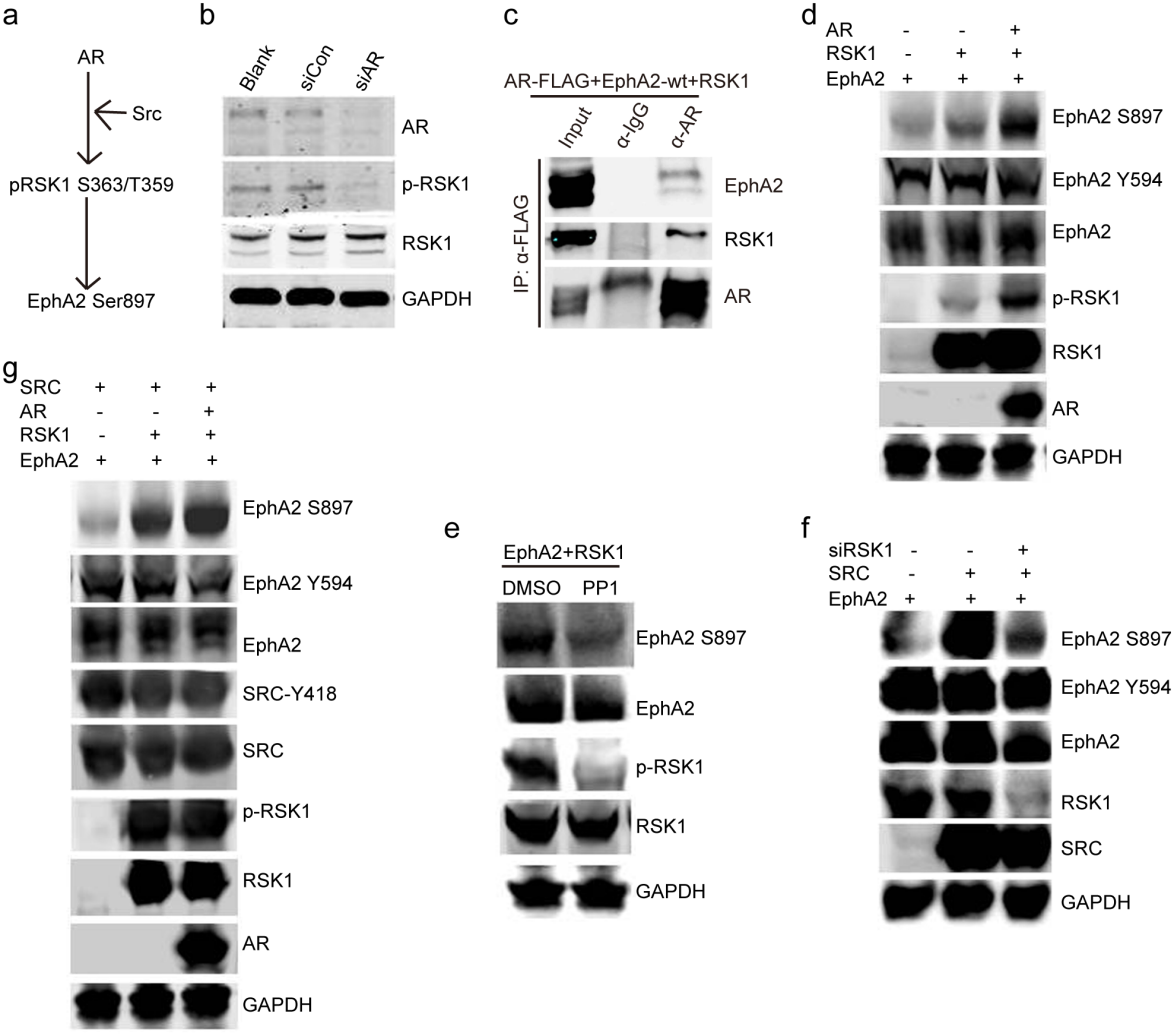
AR activates RSK1, the kinase that phosphorylates EphA2 at Ser897, by recruiting Src. (a) Schematic demonstration of AR-mediated upregulation of the phosphorylation of EphA2 at Ser897. (b) AR inhibition leads to reduced activation of RSK1. siRNA-transfected cells were subjected to western blotting. (c) The interaction between AR and RSK1 was verified. Plasmids expressing FLAG-tagged AR and RSK1 were transfected into 293T cells. 48 h later, the cells were subjected to immunoprecipitation using anti-FLAG M2 affinity beads, followed by western blotting. (d) Ectopic AR further promotes the expression of pEphA2 Ser897 upregulated by RSK1. Expression plasmids were transfected into basic medium-cultured 293T cells in the indicated combinations. (e) Expression of phosphorylated RSK1 is considerably impaired by the Src inhibitor PP1. 293T cells were cultured, and then co-transfected with plasmids expressing GFP-tagged EphA2 and hemagglutinin-tagged RSK1 for 48 h prior to the application of PP1 (at 5μM) for 4 h. (f) RSK1 is required by Src to activate the pEphA2 Ser897. siRNA targeting RSK1 and the control siRNAs were transfected into basic medium-cultured 293T cells for 24h, followed by transfection of expression plasmids in indicated combinations. After 36 h, cells were harvested. (g) Src andRSK1 upregulate the phosphorylation of EphA2 at Ser897, and the effect is mediated by the AR. Expression plasmids were transfected into basic medium-cultured 293T cells in the indicated combinations. Each reaction was repeated in, at least, triplicate.

Next, we explored the molecular mechanism by which AR activates EphA2. We speculated that RSK1 may be involved in this process because it phosphorylates EphA2 at Ser897 [37, 38] (Fig 7a). It was demonstrated that RSK1 is a critical downstream signaling component of the AR, as indicated by the near elimination of RSK1 activation by the AR siRNA, compared with control treatments (Fig 7b). We further confirmed that AR forms complex with RSK1 in ectopic expressed 293T cells by co-IP assay (Fig 7c). This regulation leads to the dramatically increased expression of the pEphA2 Ser897 resulting from co-transfection of the recombinant RSK1 plasmid with ectopic AR (Fig 7d). As Src acts as an upstream signal to directly phosphorylate RSKs [37–39], we hypothesized that the AR may activate RSK1 via Src. We verified that treatment with PP1, a Src inhibitor, reduced the level of phosphorylated RSK1 and, consequently, downregulated the phosphorylation of pEphA2 Ser897, without affecting the total level of phosphorylation (Fig 7e). The effectiveness of PP1 was validated by its ability to completely inhibit Src phosphorylation (S6c Fig). The diminishment of the phosphorylation of pEphA2 Ser897 by RSK1 siRNAs further indicated the requirement for RSK1 in Src-mediated activation of EphA2 (Fig 7f). The results further showed that the remarkable enhancement of EphA2 Ser897 phosphorylation, which was induced by ectopic Src and RSK1, was further promoted by co-transfection with an AR-expressing plasmid (Fig 7g).

**Fig 8:**
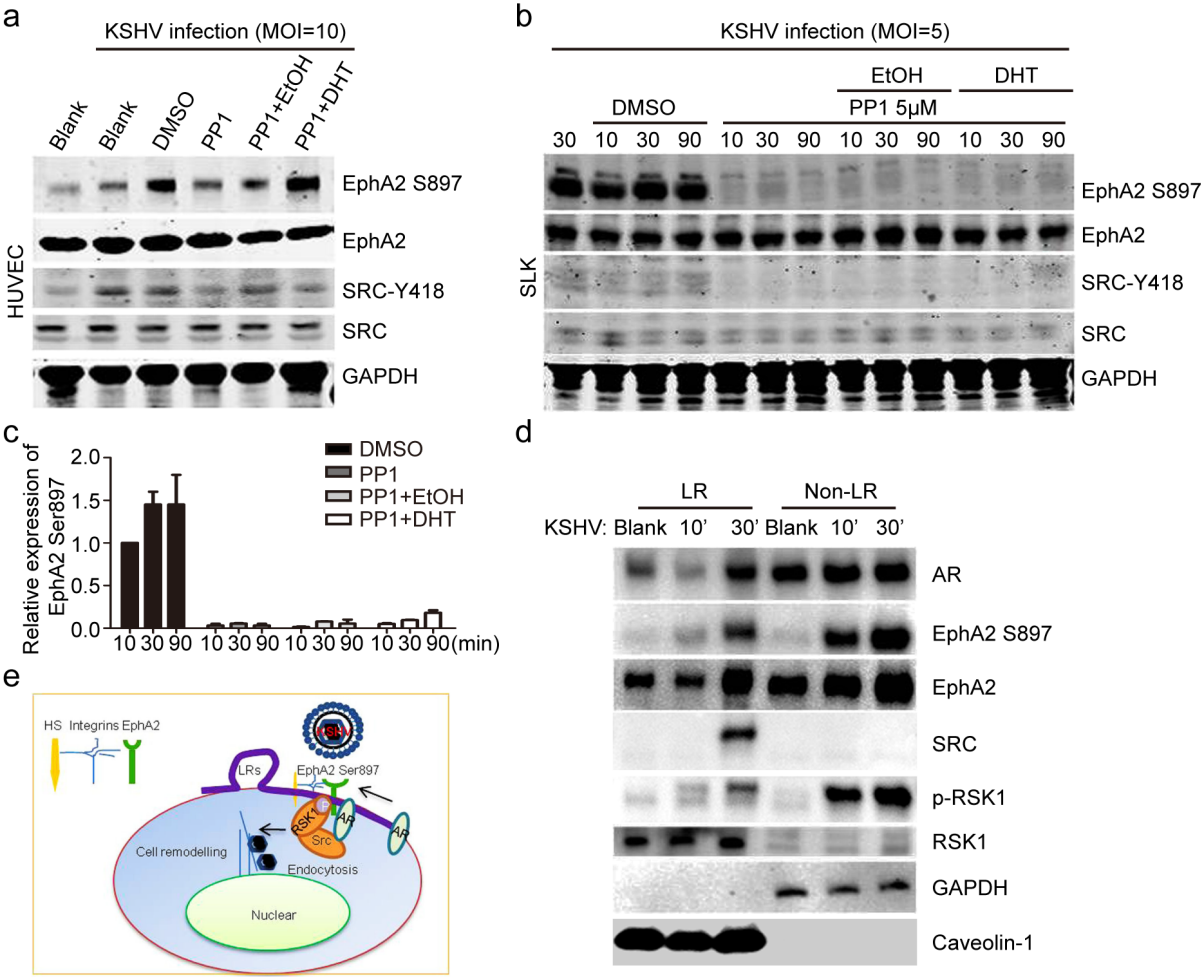
AR-induced downstream signaling cascades were recapitulated in membrane-LRs of HUVECs cells, upon KSHV infection. (a, b) PP1-induced decreasing expression of pEphA2 at Ser897 is rescued by DHT treatment upon KSHV infection. HUVECs (a) and SLK cells (b) were treated with DHT at 1 uM or 3uM or ethanol (at 0.1 or 0.3% v/v) for 24 h, followed by serum-starvation for 6 h. The PP1 inhibitor (at 5 μM) was applied to cells for 4 h, followed by inoculation with KSHV for the indicated times. Each reaction was repeated in, at least, triplicate. (c) The densitometry histogram representing the percentage of EphA2 that is phosphorylated at Ser897 from each group in (b) is shown. The fold-increase in the level of EphA2 that is phosphorylated at Ser897 for each treatment was measured by calibrating it to the corresponding level of actin expression, and then normalizing it to the level in untreated cells. Data are shown as the mean±SEM; n = 3. One-way ANOVA analysis was performed. (d) Membrane AR and AR-induced Src/pRSK1/pEphA2 Ser897 were upregulated by KSHV infection in LR fragments of HUVECs cells. Cells were left uninfected or infected with KSHV at MOI = 10 for the indicated times, and then subjected to isolation of lipid rafts and immunoblot detection. (e) The working model of AR-mediated phosphorylation of EphA2 at Ser897 during KSHV entry.

More importantly, all of these regulatory events were recapitulated during a KSHV infection (Fig 8a and 8b). Compared with ethanol treatment, the dramatic inhibition of EphA2 Ser897 phosphorylation by PP1 was significantly rescued by DHT treatment, both in HUVECs (Fig 8a) and SLK cells (Fig 8b). More specifically, EphA2 Ser897 phosphorylation was moderately restored by DHT in SLK cells, except at 90 min p.i. (Fig 8b), while much stronger restoration occurred in HUVECs. The densitometry values that reflect the level of EphA2 phosphorylation at Ser897 (Fig 8b) are provided in Fig 8c. Intriguingly, these regulatory events of AR-induced signal pathways were observed in the LR fraction by membrane raft isolation in HUVEC cells (Fig 8d). Upon KSHV infection, the greatly increased expression of AR, along with Src, was specifically identified in cell membrane compartments of HUVEC cells, while extensive upregulation of pEphA2 Ser897 and pRSK1 occurred throughout whole cell lysate (Fig 8d). In summary, we propose that the membrane-localized AR is the major component that mediates the phosphorylation of KSHV EphA2 by associating with Src, and that the Src-recruited RSK1 kinase phosphorylates EphA2 at Ser897, which is required for successful KSHV entry (Fig 8e).

## Discussion

Sex-based differences result in different immune responses and disease susceptibilities, which lead to a male predominance for certain infectious diseases. However, the mechanisms for this phenomenon remain largely unknown [1–5]. The male predominance of KS in clinical practice has also been well documented [13–18]; however, its mechanism is not well understood either. Herein, to our knowledge, for the first time we demonstrated a mechanism by which male hormones act as a host factor to facilitate KSHV entry by mediating Src/RSK1/EphA2 Ser897 signaling cascades, which implies a novel mechanism for gender disparity in KS.

KSHV infection is essential for the development of KS. In the present study, we demonstrated that male sex steroids facilitated the very early step of KSHV infection. Although the vast majority of KS spindle cells are latently infected with the virus, in a small proportion of infected cells the virus undergoes lytic replication leading to the production of mature virus [14, 17]. Herein, we postulate a novel role of male hormones as internal stimuli that facilitate this secondary infection which may contribute to the pathogenesis.

In contrast to direct binding to HBV genome of AR, and promotion of viral replication [10, 11], our findings provide the first evidence that membrane-localized AR is exploited for KSHV entry and endocytosis. Although intracellular AR is conventionally recognized as a transcription factor, in the last decade, studies have shown that the actions of androgens are initiated through the stimulation of membrane androgen-binding sites or receptors (mARs) [23, 24, 39]. Although the molecular identity of these mARs remains elusive, their activation triggers multiple non-genomic signaling cascades, and it regulates numerous cell responses [39]. Here, for the first time, we demonstrated that this counterpart of the AR could be involved in infectious disease.

Cell entry by KSHV is a multistep process involving viral envelope glycoproteins as well as several cellular attachment and entry factors [29, 30]. One of these is EphA2, which is localized to cell membrane subdomains/LRs, and, therefore, it has great potential for crosstalk with membrane-localized AR that is distributed in these specific subcompartments [25, 26]. A lack of the intracellular kinase domain of EphA2 leads to a dramatic (greater than 70%) decrease in KSHV infection rates [25]. To our knowledge, the present study is the first to demonstrate that EphA2 phosphorylation at Ser897 is primarily responsible for this effect.

Additionally, we identified a novel mechanism of the AR/Src/RSK1 signaling cascade, accounting for EphA2 phosphorylation. Gao lab and Chandran lab demonstrated that vital host pathways ERK (Extracellular signal-Regulated Kinase)/MAPK (Mitogen-Activated Protein Kinase) and FAK (Focal Adhesion Kinase)/PI3K (Phosphoinositide 3-kinase Phosphatidylinositol-4,5-bisphosphate 3-kinase) /PKC (Protein kinase C) are essentially required for KSHV primary infection [40–42]. RSKs are downstream effectors of the Ras-ERK/MAPK signalling cascade [38], thus these pathways could also be alternative mechanisms for AR-mediated promotion of KSHV infection. As EphA2 had been identified as KSHV receptor for endothelial cells [25], it turned out to be the major candidate by male sex steroids in our first attempt. KSHV utilizes heparan sulfate, integrins, xCT (Cystine Transporter) and DC-SIGN (Dendritic Cell Specific Intracellular adhesion molecule-3 (ICAM-3) Grabbing Non- integrin) in context of target cell types [43], therefore they could also be hijacked by AR and would be interesting for future study.

Upon KSHV infection, it is notable that besides in LRs fragments, an immediate early accumulation of cytosolic pEphA2 Ser897 and pRSK1 are observed and it may be due to their roles in versatile biological processes other than receptor activation. EphA2 and FAK/Src/PI3K/RhoGTPase pathogenically function in cell cytoskeleton remodeling by providing the mechanical force necessary to complete endocytosis [26, 31], suggesting that cytoplasmic RSK1 may participate in the process as well. The phosphorylation of EphA2 at Ser897 is also exploited by *Chlamydia trachomatis* to activate phosphatidylinositol-4,5-bisphosphate 3-kinase (PI3K) signaling to induce apoptosis resistance [44]. Although the phosphorylation of EphA2 at Ser897 has been previously reported to function in the ligand-independent promotion of tumor malignant progression [37, 38, 45], its role in infectious diseases need further investigation. Therefore, it may represent a new candidate for drug development for the prevention of KSHV infections, at least in high-risk populations.

Male hormones contribute to the male predominance in certain infectious diseases through various mechanisms, either having an indirect function by hijacking immune cells [4–8], or by physical interaction with pathogens [10–11]. For the first time, this study demonstrated that the male sex hormones acted as host cofactors in the pathogenesis of primary KSHV infection, which implies a novel mechanism for gender disparity in KS. Considering that EphA2 is also the receptor for some other viruses such as hepatitis C virus (HCV) and that it is a signaling hub [36, 46], our findings may be relevant to other viral diseases and to endocrine-associated oncogenesis.

## Materials and methods

### Cell lines, antibodies, reagents, and plasmids

HUVECs (ATCC CRL-1730) were cultured in complete endothelial basal medium-2 (Lonza). LEC were purchased from PromoCell (C-12216) and cultured with Endothelial Cell Growth Medium MV2 kit (C-22121, PromoCell). BJAB (KSHV-negative B cells) and BCBL1 (KSHV-positive PEL cells) were generously provided by Dr. Erle S Robertson (University of Pennsylvania, USA) and were maintained in Roswell Park Memorial Institute 1640 medium (HyClone) supplemented with 10% FBS (HyClone). KS-derived SLK epithelial cell lines and doxycycline inducible recombinant KSHV.219 harboring SLK (iSLK.219) cell lines was established by J. Myoung and D. Ganem, and was kindly provided by Fanxiu Zhu (Florida State University). iSLK.219 cells were cultured in DMEM supplemented with 10% fetal bovine serum, 1% penicillin-streptomycin, 1 μg/ml puromycin, 250 μg/ml G418, and 1 mg/ml hygromycin B. Androgen-sensitive human prostate adenocarcinoma cells (LNCap) (TCHu173), androgen-independent prostate cancer cells (PC3) (TCHu158) and 293T cells (GNHu17) were purchased from cell bank/stem cell bank of Shanghai Institutes of Biological Sciences, Chinese Academy of Sciences (Shanghai, China). SLK, LNCap, PC3 and 293T cells were maintained in Dulbecco’s modified Eagle’s medium (DMEM) (HyClone) supplemented with 10% fetal bovine serum (FBS) (HyClone). Before DHT treatment, charcoal-stripped FBS (10%; CD-FBS) (Sigma–Aldrich, St. Louis, MO, USA) in basic medium was pre-utilized for cell culture for 24 h, from which endogenous hormones and growth factors had been removed.

The antibodies and reagents were as follows: anti-AR antibody (ab74272, Abcam),anti-EphA2 antibody(ab54968, Abcam), anti-phospho-EphA2 (Y594) (3970S, Cell Signaling), anti-phospho-EphA2 (Y596/602) (92590, Millipore), anti-phospho-EphA2 (S897) (6347S, Cell Signaling), anti-Src pan antibody (44656G, Invitrogen), anti-phospho-Src antibody (S418) (44660G, Invitrogen), anti-phospho-RSK1 antibody (T539+S363) (Cy5344, Abways), anti-RSK1 antibody (ab32526, Abcam), and anti-KSHV ORF8 antibody (ab36599, Abcam), anti-LANA monoclonal antibody (LN53, ABI); anti-EEA1 antibody (ab2900, Abcam), anti-LANA antibody(1B5) was prepared in our laboratory. Secondary antibodies (Thermo Fisher Scientific) included goat anti-rabbit antibodies conjugated with Alexa Fluor 488 [A-11094], 555 [A27017], and 680[A27020]), and goat anti-mouse antibodies conjugated with Alexa Fluor 488 [A-11001], 555 [A-21422], and 680 [A-28183]).

DHT(D-073, Sigma–Aldrich), PP1 (sc-203212, Santa Cruz Biotechnology, Dallas, TX, USA), Protease Inhibitor Cocktail Set III (539134, Millipore), Phosphatase Inhibitor Cocktail (sc-45044, Santa Cruz Biotechnology), doxycycline hyclate(D9891-25G-9, Sigma–Aldrich), hygromycin (V900372-1G, Sigma–Aldrich), puromycin (OGS541-5UG, Sigma–Aldrich), TPA (P1585, Sigma-Aldrich) and G418 disulfate salt (A1720-5G, Sigma–Aldrich); control siRNA (fluorescein isothiocyanate conjugate)-A (sc-36869, Santa Cruz Biotechnology), AR siRNA (sc-29204, Santa Cruz Biotechnology), EphA2 siRNA (sc-29304, Santa Cruz Biotechnology), and RSK1 siRNA (6309S, Cell Signaling Technology); Lipofectamine 2000 (11668019, Thermo Fisher Scientific), 4',6-diamidino-2-phenylindole (DAPI) (Beyotime, c1002); anti-FLAG M2 affinity gel (A2220-5 ml, Sigma-Aldrich), recombinant protein A/G agarose (15948-014/15920-010, Invitrogen), glutathione Sepharose 4B (17-0756-01, GE Healthcare); Vybrant LR Labeling Kits (Life Technologies, v-34404), Caveolae/Rafts Isolation Kit (Sigma, CS0750), the TNT T7 Quick Coupled Transcription/Translation System (L1170, Promega), the Accuprep Genomic DNA Extraction Kit (k-3032, Bioneer), the Mut Express II Fast Mutagenesis Kit V2 (C214-01, Vazyme), Amicon Ultra-4 Centrifugal Filter Units (Millipore, UFC801008) and collagen type I cell ware coverslips (354089, BD Biosciences).

Plasmids: The plasmids pAR-FLAG (expressing FLAG-tagged AR), pEphA2-copGFP (expressing EphA2), and pRSK1-HA (expressing hemagglutinin-tagged RSK1) comprising amino acids 512–918 (ref [M23263.1] for AR) or full length sequences (ref[NM_004431.3] for EphA2 and ref[EF043873.1] for RSK1)were generated by PCR amplification of the respective fragment from cDNAs. The resulting amplicons were inserted into the pCDH-CMV-SF-IRES-Blast, pCDH-CMV-MCS-EF1-copGFP (System Biosciences, SBI), and pCMV-HA vectors (Clontech), respectively. The plasmid pSRC-FLAG (expressing FLAG-tagged SRC) comprising full length sequence (ref [NM-005417] for SRC) was generated by PCR amplification of a target fragment from SRC expressing bacteria (X-GWDD70769, Genechem). AR-pcDNA3.1(+)-HA was constructed by subcloning the HA-AR fragment into pcDNA3.1(+) from pHA-AR which was constructed by subcloning the AR fragment from pAR-FLAG into the pCMV-HA vector. Three truncations of EphA2 were fused to GST in the pGEX-4T-1 backbone vector (GE Healthcare), and they comprised amino acids 1–519 (the extracellular domain),1–558 (the extracellular plus transmembrane region), and 613–871 (the kinase domain). The EphA2 Ser897Asn mutant was obtained by site-directed mutagenesis of the pEphA2-copGFP plasmid. The reporter plasmid pLANA-pGL2.0 was described previously. The plasmid pHSV1-UL30-C comprising part of the C-terminal of HSV1-UL30 (NC_001806, 65581–66480) was generated by PCR amplification of the according fragment from HSV1 genome and inserted into the pCDH-CMV-SF-IRES-Blast. All of the primers are summarized in S1 Table.

### Immunofluorescence assay

Cells were fixed with 4% paraformaldehyde for 30 min at room temperature, permeabilized with 0.5% Triton X-100, and blocked with 20% normal goat serum (Life Technologies), and then they were reacted with the indicated antibodies, followed by fluorescent dye-conjugated secondary antibodies (1:1,000 dilution). The dilution factor for individual primary antibodies was generally 1:200. Cell nuclei were stained with DAPI LR labeling was performed according to the manufacturer’s recommendation before the fixation. Briefly, live cells were incubated with the fluorescent cholera toxin B subunit (CT-B) conjugate (1:1,000 dilution), followed by crosslinking with the anti–CT-B antibody (1:200 dilution). The procedures were performed at temperatures below 4°C using chilled complete growth medium. Coverslips were mounted with anti-fade mounting medium (Beyotime) and photographed using a digital camera and software (Olympus FV-1200).

### KSHV virion purification and primary infections

Recombinant KSHV.219 (rKSHV.219) stocks and wild-type virions were acquired by inducing iSLK-BAC16 cells with doxycycline and inducing BCBL1 cells with 12-O-tetradecanoyl phorbol-13-acetate (TPA) individually, as described previously [25, 26]. Briefly, five days later, the supernatant was collected and cleared of cells and debris by centrifugation (1500 g for 1 h at 4°C) and 0.45 um syringe filtration. Virus particles were pelleted by ultracentrifugation (25,000 × *g* for 2 h at 4°C) using a SW28Ti rotor. Various amounts of cell-free virus supernatants were diluted and inoculated into 293T cells that were seeded at approximately 5×10^5^ cells/well into six-well plates 24 h prior to infection. Following inoculation, the plates were immediately centrifuged (660 g for 2 h at 30°C) and then placed back into the CO_2_ incubator. After the centrifugation, the inoculum was removed and replaced with fresh medium. Cells were collected 24 h later and washed once with cold phosphate-buffered saline (PBS). The percentage of GFP-positive cells was determined using a LSRII fluorescence-activated cell sorter (BD Biosciences). Layout and mean fluorescence parameters were analyzed using FlowJo v4.5.9 software (FLOWJO, LLC). And DNA numbers for wild-type KSHV were determined by LANA amplification in quantitative qRT-PCR analysis. The multiplicity of infection (MOI) was expressed as the number of GFP-positive cells and the normalized LANA expression in each well at the time of analysis. For the low production of KSHV in BCBL1 cells, wild-type virions were only used in immunofluorescent detection for LANA expression. Neither GFP nor RFP of rKSHV.219 can be detected at 10 to 30 minutes p.i., thus the recombinant virus was used for immunofluorescent analyzing to KSHV entry.

During KSHV infections, different amounts of concentrated virus were added to HUVECs, and SLK and 293T cells at MOIs of 10, 5, and 1, respectively. The inoculation were replaced with the corresponding fresh medium, and the cells were cultured for the indicated times. After removing viruses by washing twice with PBS, the cells were prepared under the indicated conditions and subjected to the following conditions.

HUVECs and SLK cells were infected with KSHV for 1 h at 4°C for virus binding and at 37°C for virus entry. Cells were washed twice with PBS to remove unbound viruses, and they were subjected to an additional treatment with 0.25% trypsin-EDTA for 5 min at 37°C to remove bound, but non-internalized, viruses for the virus entry analysis.

### Quantification of KSHV DNA levels and RNA transcriptions in cells

KSHV DNA was extracted according to the manufacturer’s instructions (the Accuprep Genomic DNA Extraction Kit, Bioneer). A total of 200 ng of DNA from each sample was used in a real-time DNA PCR using KSHV LANA gene-specific primers. The LANA gene cloned into the pGL2.0 vector (Promega) was used as the external standard. Known amounts of the LANA plasmid were used in the amplification reactions along with the test samples. Cycle threshold values were used to generate a standard curve and to calculate the relative copy numbers of viral DNA in the samples. The amount of KSHV DNA was normalized to the amount of purified cellular DNA as determined by primers targeting the glyceraldehyde 3-phosphate dehydrogenase gene.

Cells were lysed in TRIzol buffer (Life Technologies), and RNA was isolated according to the manufacturer’s instructions. Reverse transcription was performed with a cDNA Reverse Transcription Kit (Toyobo). Real-time reverse transcription-PCR was performed with a SYBR green Master Mix kit (Toyobo). Relative mRNA levels were normalized to the level of actin mRNA and calculated by the ΔΔCT method. The primer sequences are summarized in S1 Table.

### Cell transfection

HUVECs and SLK cells were seeded into six-well plates and transfected at ~80% confluency with siRNA pools from Santa Cruz Biotechnology targeting either the AR or EphA2. siRNAs were transfected using Lipofectamine 2000 (Thermo Fisher Scientific) according to the manufacturer’s instructions. The concentrations for HUVECs and SLK cells were 100 nM and 200 nM, respectively. Cells were cultured at 37°C for 6 h, washed, and maintained for another 18 h. siRNA targeting RSK1 was transfected into 293T cells using Lipofectamine 2000. The concentration of siRSK1 was 150 nM. Recombinant expression plasmids were transfected into 293T cells using polyethyleneimine for 12 h, and cells were continually cultured in fresh medium for 36 h before collection.

### Immunoblotting

Cell lysates were prepared in radioimmunoprecipitation assay (RIPA) buffer (50 mM Tris-HCl [pH 7.4], 150 mM NaCl, 0.5% Triton X-100) containing protease and phosphatase inhibitors. Proteins were separated by sodium dodecyl sulfate-polyacrylamide gel electrophoresis (SDS-PAGE) and transferred to polyvinylidene difluoride membranes for immunoblotting with the indicated antibodies.

### Co-immunoprecipitations and GST pulldowns

Cells were lysed in RIPA buffer containing protease and phosphatase inhibitors for 2 h on ice, with brief vortexing every 10 min. The lysates were centrifuged at 15,000 g for 20 min at 4°C to remove cell debris. Supernatants were incubated with the indicated antibodies or affinity beads at 4°C for 2 h, with gentle rotation. The immunoprecipitates were separated by SDS-PAGE and analyzed by immunoblotting.

GST fusion proteins were expressed in *Escherichia coli* BL21 and purified using glutathione-Sepharose 4B (GE Healthcare) according to the manufacturer’s instructions. For the pulldown assays, glutathione beads were incubated with purified GST-tagged proteins in RIPA buffer containing 0.5% bovine serum albumin at 4°C overnight, with gentle rotation. In vitro-translated AR protein, which was produced by the TNT coupled transcription/translation system (Promega), was further incubated for 2 h. Bound proteins were analyzed by SDS-PAGE and immunoblotted with an anti-HA antibody.

### Isolation of caveolae/rafts

2×10^7^ of HUVEC cells at 80–90% confluence were infected by KSHV at an MOI of 10 for 10' and 30', or left uninfected, and were washed twice with ice-cold PBS, then subjected to the isolation of the microdomains from the cell plasma membrane according to the manufacturer’s instructions (CS0750, Sigma). All the work was performed in a cold room. Briefly, a cell lysate was prepared by adding lysis buffer containing Triton X-100 and incubating on ice for 1h. Density gradients at 0%, 20%, 25%, 30% and 35% were prepared using the recommended amounts of the cell lysate, lysis buffer and OptiPrep, and then centrifuged at 200,000 *g* using a SW28Ti rotor (CP80NX, HITACHI) for 4h at 4°C. Each fraction was carefully collected from the top to the bottom of the ultracentrifuge tubes. The LR subcompartment (at 20% and 30% OptiPrep layers) fractions were condensed using a centrifugal filter (Millipore Amicon Ultra, UFC801008) and were detected by immunoblot assay.

### Statistics

Data are expressed as means ± standard errors of the means (SEM). One-way ANOVA analysis, paired and unpaired Student’s *t*-tests were performed with GraphPad Prism software (GraphPad Software, Inc., 7825 Fay Avenue, Suite 230, La Jolla, CA 92037 USA).

## Supporting information

S1 Fig (a) The full-length isoform of AR was abundantly expressed in androgen-sensitive LNCap cells, but not in the non-sensitive PC3 cells. Cells were cultured in complete medium and prepared for WB assay. (b) The immunofluorescence staining of the AR in cell nucleus is specific, as indicated by the much stronger signals from LNCap cells than from PC3 cells. Cells were cultured in complete medium and prepared for immunofluorescence detection. Scale bars represent 50 μm. Representative images are shown. Each reaction was repeated in, at least, triplicate.
(TIF)

S2 Fig Androgens promote KSHV primary infection in LECs cells. (a) DHT treatment increases AR expression in LECs cells. Cells were treated by increasing the amount of DHT or its solvent, ethanol, for 24 h, and subjected to immunoblotting with the indicated antibodies. Charcoal-stripped FBS was used for cell culture. (b) The expression of AR target genes increases in response to DHT treatment in LECs cells. Cellular RNA was prepared from the same samples, and the mRNA expression of the NCOA2 and POV1 genes was determined by normalization to GAPDH gene expression and then compared with untreated cells. (c, d) DHT treatment increases the KSHV genome copy number and the transcription of viral genes. Cells were treated as described above and infected for 24 h with KSHV at an MOI of 10 for LECs cells. The extraction of total DNA and RNA and the following analysis were performed as previously described. (e, f) DHT treatment considerably increases LANA-positive nuclear staining in LECs cells. The same treatment and quantitative analysis were performed to LECs cells as above. Scale bars represent 10 μm. Fifty cells from each image were randomly selected and the quantitative analysis to fluorescence density was performed as mentioned above. Data are shown as the mean±SEM; n = 3. One-way ANOVA analysis was performed on (b-d) and f. * p,0.05, ** p,0.01, *** p, 0.001, ^n.s.^ p, no significance.
(TIF)

S3 Fig The contaminant green or red fluorescent signals from rKSHV.219 virus cannot be detected during KSHV entry. HUVEC cells were infected by KSHV at MOI = 10 for indicated times, from 10 minutes to 48 hours, or left uninfected. At the time points, cells were harvested and immunofluorescently analyzed as previously described. Alternatively, cells were mock stained by only adding dilution buffer of α-gB and α-AR antibody, with no change to other procedures. Scale bars represent 50 μm. Representative images are shown. Each reaction was repeated in, at least, triplicate.
(TIF)

S4 Fig (a) Typical LRs labeling was identified in KSHV-uninfected and infected B cells. BJAB and BCBL1 cells were cultured and subjected for immunofluorescent detection as described previously. (b) The co-localization between KSHV gB and early endosome marker EEA1 was verified at early stage of KSHV endocytosis. HUVECs were starved without serum for 6 h and infected by KSHV at MOI = 10 for 20’. Cells were subjected for immunofluorescent detection as described previously. (c, d) Inhibition of AR and EphA2 expression did not affect HSV1 binding and entry in HUVECs (c) and SLK cells (d). siRNA-transfected cells were infected by HSV1 for 1 h at 4°C or 37°C with gentle shake every 15 min. After washing, total DNA was isolated and subjected to real-time DNA PCR of the UL30 gene. For virus entry detection, an extra 0.25% trypsin-EDTA treatment for 5 min at 37°C, after washing with PBS, was used to remove bound, but not internalized, viruses. (e, f) DHT treatment promotes virion accumulation around the cell nucleus in both HUVECs and LECs cells. Cells were treated with DHT or ethanol for 24 h, followed by inoculation with KSHV for 20 min. After removing unbound viruses, the cells were processed for immunofluorescence analyses using the indicated antibodies. Charcoal-stripped FBS was used for cell culture. Representative images are shown. Each reaction was repeated in, at least, triplicate. Data are shown as the mean±SEM; n = 3. One-way ANOVA analysis was performed on (c) to (d). ^n.s.^ p, no significance.
(TIF)

S5 Fig (a) The functional translocation of EphA2 that is phosphorylated at Ser897 into the nucleus is facilitated by DHT treatment. HUVECs were left untreated or treated with DHT or ethanol for 24 h, and then subjected to immunofluorescence analyses using the indicated antibodies. (b) KSHV infection further promotes the above translocation upon DHT treatment. HUVECs were left untreated or treated by DHT as described above, followed by KSHV infection for 20 min. Cells were washed, fixed, and processed for immunofluorescence with the indicated antibodies. Charcoal-stripped FBS was used for cell culture. Each reaction was repeated in, at least, triplicate. Representative images are shown. (c) DHT treatment increases the level of EphA2 phosphorylation at Ser897 without KSHV infection. SLK cells were left untreated or treated by DHT or ethanol for 24 h, and cell lysates were prepared in the presence of protease and phosphatase inhibitors, followed by western blotting using the indicated antibodies. Charcoal-stripped FBS was used for cell culture.
(TIF)

S6 Fig (a) Images from Fig 5c were analyzed using FV10-ASW3.1 viewer (Olympus). Cells were separated by polygon and the intensity of the red fluorescence was analyzed by the software. Representative images are shown. Each reaction was repeated in, at least, triplicate. (b) The AR co-localizes with Src throughout the entirety of androgen-sensitive LNCap cells. Representative images are shown. (c) PP1 treatment specifically reduces the phosphorylation of EphA2 at Ser897, independently of KSHV infection. SLK cells that were cultured in basic medium were left untreated or treated with PP1 or dimethyl sulfoxide for 4 h prior to cell harvesting. Cell samples were prepared as described above and subjected to western blotting with the indicated antibodies.
(TIF)

S1 Table Primers for cDNA cloning and qPCR analysis. (DOCX)
