# Supplementary figures and images for: Male hormones activate EphA2 to facilitate Kaposi’s sarcoma-associated herpesvirus infection: Implications for gender disparity in Kaposi’s sarcoma
Source: PLoS Pathog. 2017 Sep 28;13(9):e1006580. doi: 10.1371/journal.ppat.1006580 (PMC5619820; doi:10.1371/journal.ppat.1006580)

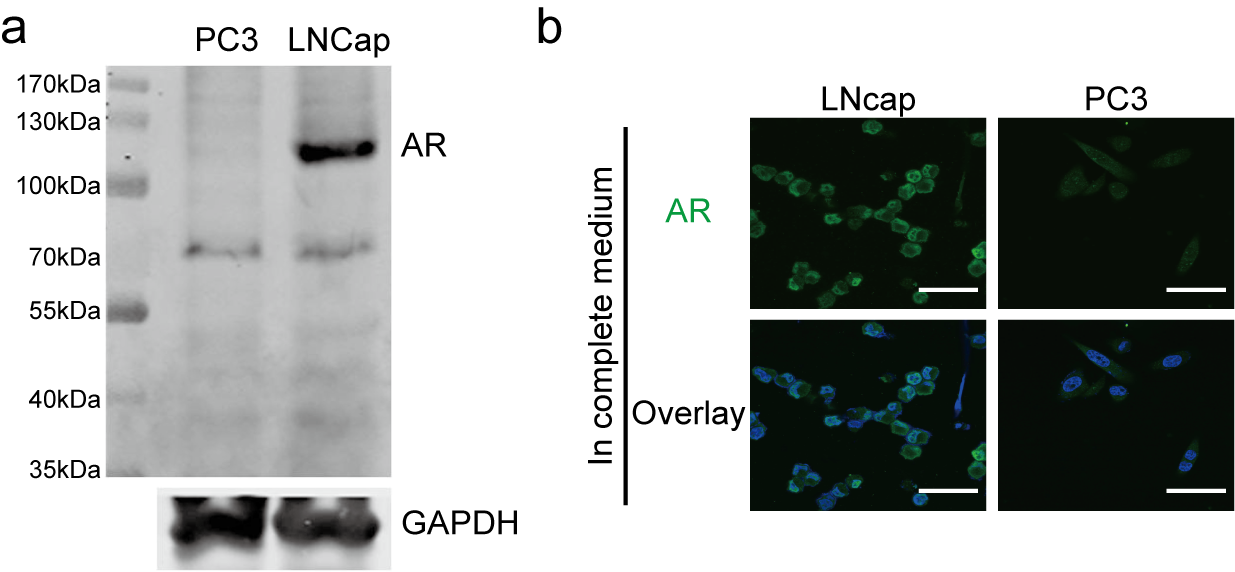

Supplement: S1 Fig — (a) The full-length isoform of AR was abundantly expressed in androgen-sensitive LNCap cells, but not in the non-sensitive PC3 cells. Cells were cultured in complete medium and prepared for WB assay. (b) The immunofluorescence staining of the AR in cell nucleus is specific, as indicated by the much stronger signals from LNCap cells than from PC3 cells. Cells were cultured in complete medium and prepared for immunofluorescence detection. Scale bars represent 50 μm. Representative images are shown. Each reaction was repeated in, at least, triplicate. (TIF) [file ppat.1006580.s001.tif]

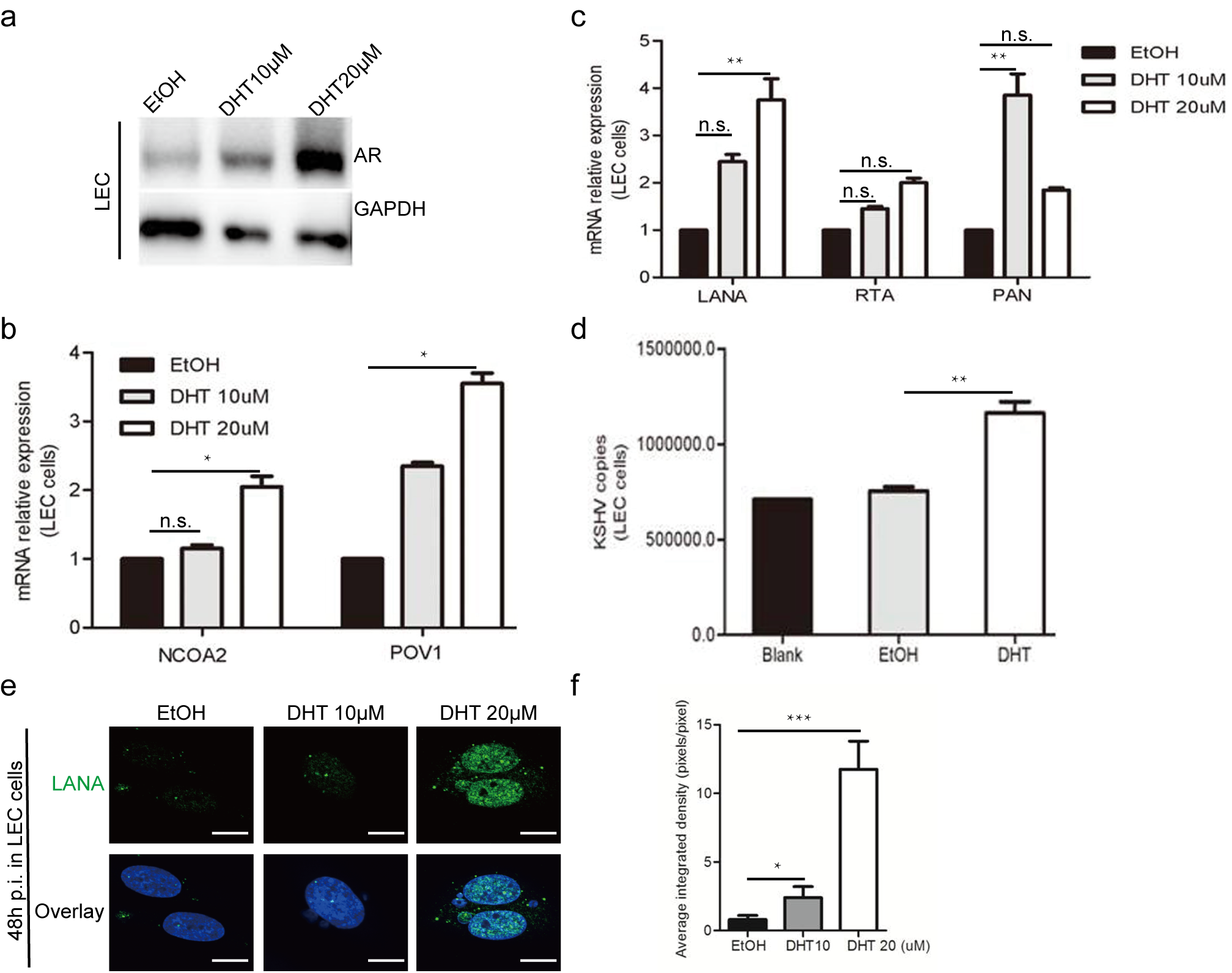

Supplement: S2 Fig — (a) DHT treatment increases AR expression in LECs cells. Cells were treated by increasing the amount of DHT or its solvent, ethanol, for 24 h, and subjected to immunoblotting with the indicated antibodies. Charcoal-stripped FBS was used for cell culture. (b) The expression of AR target genes increases in response to DHT treatment in LECs cells. Cellular RNA was prepared from the same samples, and the mRNA expression of the NCOA2 and POV1 genes was determined by normalization to GAPDH gene expression and then compared with untreated cells. (c, d) DHT treatment increases the KSHV genome copy number and the transcription of viral genes. Cells were treated as described above and infected for 24 h with KSHV at an MOI of 10 for LECs cells. The extraction of total DNA and RNA and the following analysis were performed as previously described. (e, f) DHT treatment considerably increases LANA-positive nuclear staining in LECs cells. The same treatment and quantitative analysis were performed to LECs cells as above. Scale bars represent 10 μm. Fifty cells from each image were randomly selected and the quantitative analysis to fluorescence density was performed as mentioned above. Data are shown as the mean±SEM; n = 3. One-way ANOVA analysis was performed on (b-d) and f. * p,0.05, ** p,0.01, *** p, 0.001, n.s. p, no significance. (TIF) [file ppat.1006580.s002.tif]

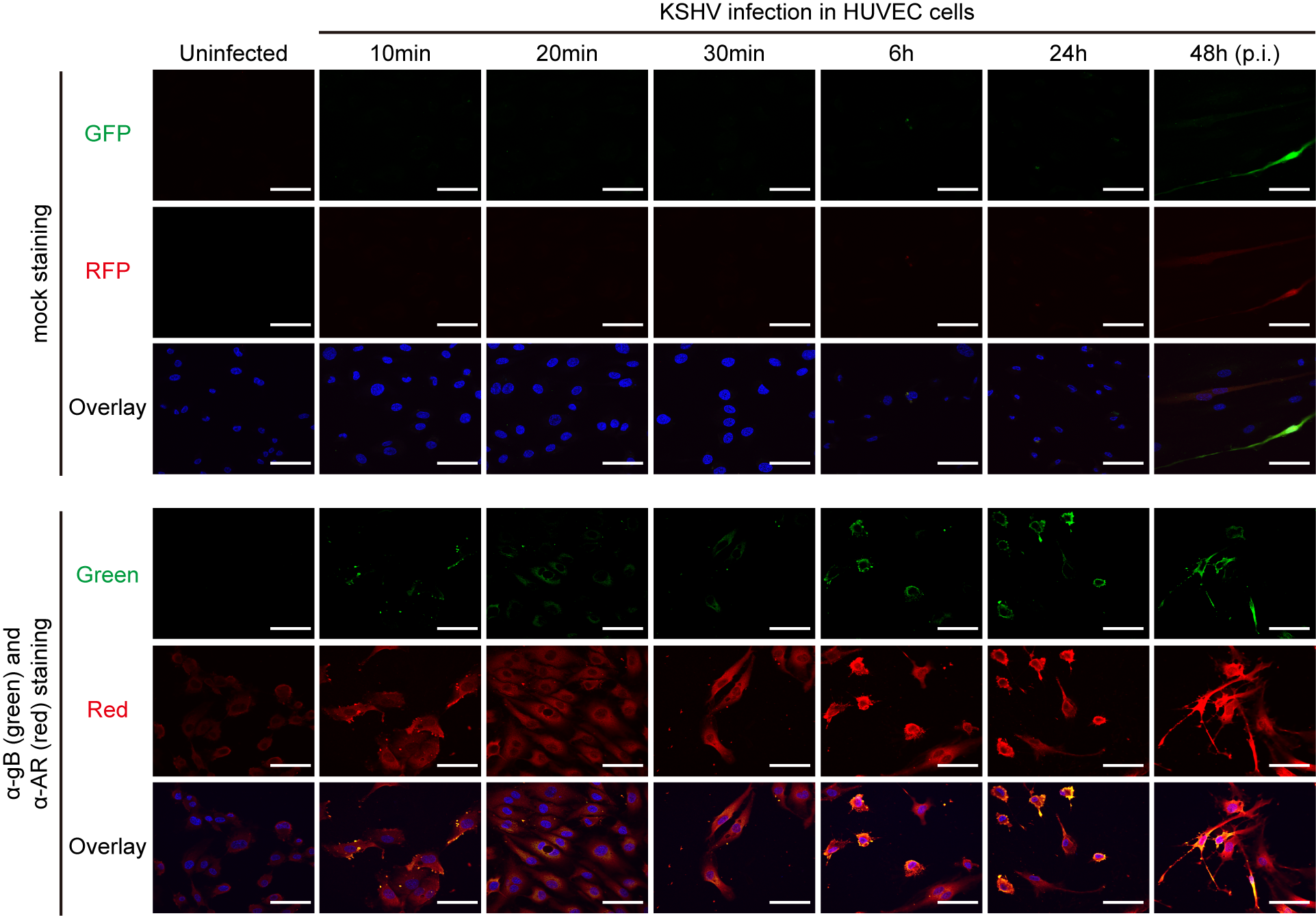

Supplement: S3 Fig — HUVEC cells were infected by KSHV at MOI = 10 for indicated times, from 10 minutes to 48 hours, or left uninfected. At the time points, cells were harvested and immunofluorescently analyzed as previously described. Alternatively, cells were mock stained by only adding dilution buffer of α-gB and α-AR antibody, with no change to other procedures. Scale bars represent 50 μm. Representative images are shown. Each reaction was repeated in, at least, triplicate. (TIF) [file ppat.1006580.s003.tif]

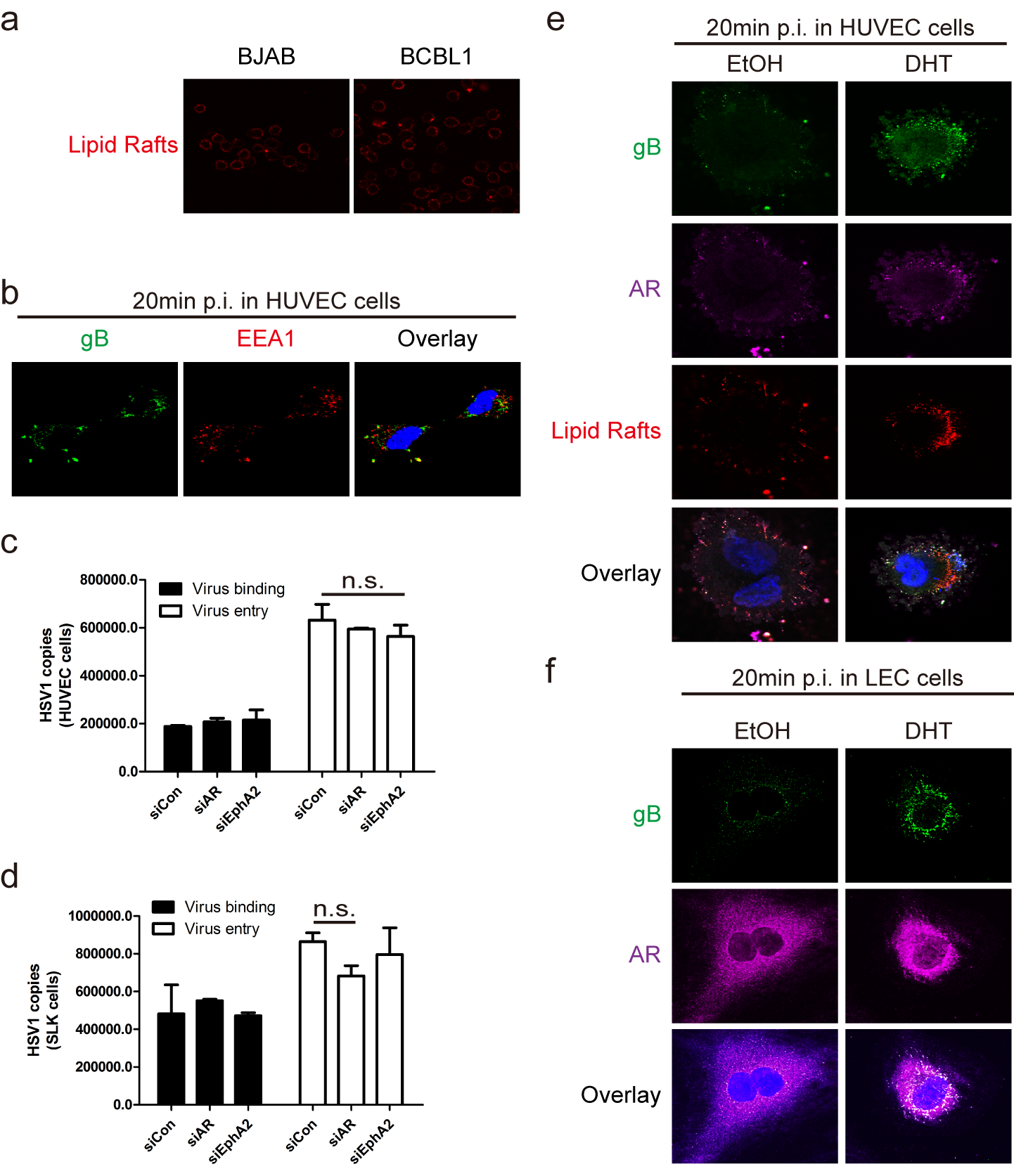

Supplement: S4 Fig — (a) Typical LRs labeling was identified in KSHV-uninfected and infected B cells. BJAB and BCBL1 cells were cultured and subjected for immunofluorescent detection as described previously. (b) The co-localization between KSHV gB and early endosome marker EEA1 was verified at early stage of KSHV endocytosis. HUVECs were starved without serum for 6 h and infected by KSHV at MOI = 10 for 20’. Cells were subjected for immunofluorescent detection as described previously. (c, d) Inhibition of AR and EphA2 expression did not affect HSV1 binding and entry in HUVECs (c) and SLK cells (d). siRNA-transfected cells were infected by HSV1 for 1 h at 4°C or 37°C with gentle shake every 15 min. After washing, total DNA was isolated and subjected to real-time DNA PCR of the UL30 gene. For virus entry detection, an extra 0.25% trypsin-EDTA treatment for 5 min at 37°C, after washing with PBS, was used to remove bound, but not internalized, viruses. (e, f) DHT treatment promotes virion accumulation around the cell nucleus in both HUVECs and LECs cells. Cells were treated with DHT or ethanol for 24 h, followed by inoculation with KSHV for 20 min. After removing unbound viruses, the cells were processed for immunofluorescence analyses using the indicated antibodies. Charcoal-stripped FBS was used for cell culture. Representative images are shown. Each reaction was repeated in, at least, triplicate. Data are shown as the mean±SEM; n = 3. One-way ANOVA analysis was performed on (c) to (d). n.s. p, no significance. (TIF) [file ppat.1006580.s004.tif]

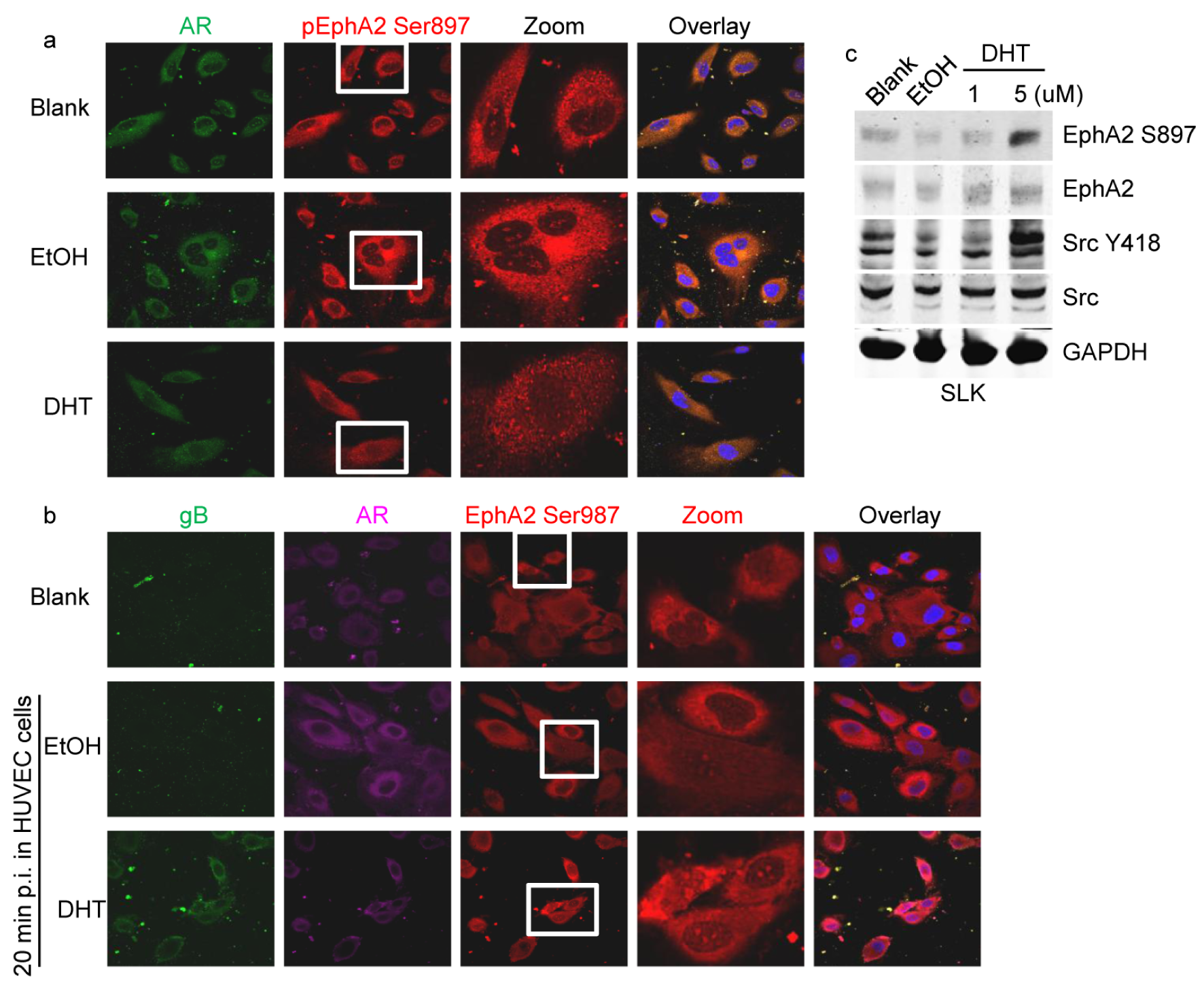

Supplement: S5 Fig — (a) The functional translocation of EphA2 that is phosphorylated at Ser897 into the nucleus is facilitated by DHT treatment. HUVECs were left untreated or treated with DHT or ethanol for 24 h, and then subjected to immunofluorescence analyses using the indicated antibodies. (b) KSHV infection further promotes the above translocation upon DHT treatment. HUVECs were left untreated or treated by DHT as described above, followed by KSHV infection for 20 min. Cells were washed, fixed, and processed for immunofluorescence with the indicated antibodies. Charcoal-stripped FBS was used for cell culture. Each reaction was repeated in, at least, triplicate. Representative images are shown. (c) DHT treatment increases the level of EphA2 phosphorylation at Ser897 without KSHV infection. SLK cells were left untreated or treated by DHT or ethanol for 24 h, and cell lysates were prepared in the presence of protease and phosphatase inhibitors, followed by western blotting using the indicated antibodies. Charcoal-stripped FBS was used for cell culture. (TIF) [file ppat.1006580.s005.tif]

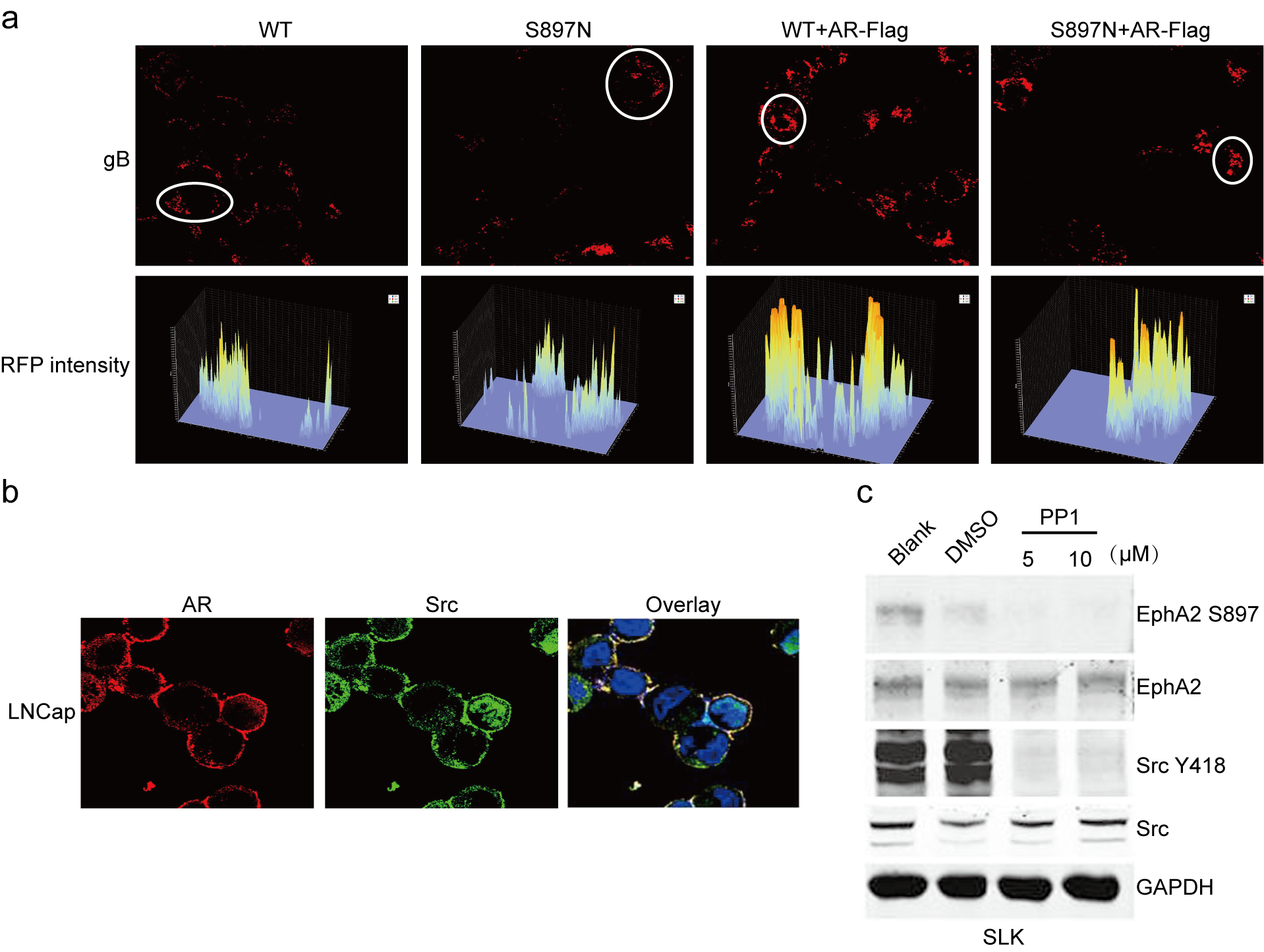

Supplement: S6 Fig — (a) Images from Fig 5c were analyzed using FV10-ASW3.1 viewer (Olympus). Cells were separated by polygon and the intensity of the red fluorescence was analyzed by the software. Representative images are shown. Each reaction was repeated in, at least, triplicate. (b) The AR co-localizes with Src throughout the entirety of androgen-sensitive LNCap cells. Representative images are shown. (c) PP1 treatment specifically reduces the phosphorylation of EphA2 at Ser897, independently of KSHV infection. SLK cells that were cultured in basic medium were left untreated or treated with PP1 or dimethyl sulfoxide for 4 h prior to cell harvesting. Cell samples were prepared as described above and subjected to western blotting with the indicated antibodies. (TIF) [file ppat.1006580.s006.tif]
