# Supplementary material for: Male hormones activate EphA2 to facilitate Kaposi’s sarcoma-associated herpesvirus infection: Implications for gender disparity in Kaposi’s sarcoma
Source: PLoS Pathog. 2017 Sep 28;13(9):e1006580. doi: 10.1371/journal.ppat.1006580 (PMC5619820; doi:10.1371/journal.ppat.1006580)
Supplement: S1 Table — (DOCX) [file ppat.1006580.s007.docx]

**S1 Table. Primers for cDNA cloning and qPCR analysis.**

| **Primers** | **Sequences (5′ to 3′)** | |
| --- | --- | --- |
| **For cDNA cloning** | | |
| pAR-FLAG F | | CAGGGGCTGGCGGGCCAGGAAAGCGACTTCACCGCACCTGATGTGTGGTACCCTGGCGGCATGGTGAGCA |
| pAR-FLAG R | | GCACTGGGTGTGGAAATAGATGGGCTTGACTTTCCCAGAAAGG |
| pHA-AR F | | CGCGTCGACTCAGGGGCTGGCGGGCCAGGAAA |
| pHA-AR R | | CGCAGATCTTCACTGGGTGTGGAAATAGATGGGC |
| AR-pcDNA3.1(+)-HA F | | CGCGGATCCCCCACCATGTACCCATACGATGTTC |
| AR-pcDNA3.1(+)-HA R | | CGCCTCGAGTCACTGGGTGTGGAAATAGATGGGC |
| pSRC-FLAG F | | CGCCTCGAGGCCACCATGGGTAGCAACAAGAGCAAGCC |
| pSRC-FLAG R | | CGCGAATTCACGAGGTTCTCCCCGGGCTGGTACT |
| pEphA2-copGFP F | | GCTCTAGAACCATGGATGGAGCTCCAGGCAGCCCGCGCCTGCTTCG |
| pEphA2-copGFP R | | CGGGATCCCCACAGTGTTCACCTGGTCCTTGAGTCC |
| pRSK1-HA F | | CGGAATTCGCCACCATGGGGATGCCGCTCGCCCAG |
| pRSK1-HA R | | CCGCTCGAGTCACAGGGTGGTGGATGGCAACTTCCT |
| pGEX-4T-1-EC-EphA2F | | ATGGAGCTCCAGGCAGCCCGCGCCTGCTTCG |
| pGEX-4T-1-EC-EphA2R | | GCTGCCGGCCCCCTGGCCCTCCTGCGT |
| pGEX-4T-1-EC-TM-EphA2F | | ATGGAGCTCCAGGCAGCCCGCGCCTGCTTCG |
| pGEX-4T-1-EC-TM-EphA2R | | GATAAAGAAGCCAACTCCTGCCAGC |
| pGEX-4T-1-KD-EphA2F | | ATGACTCGGCAGAAGGTGATCGGAGCA |
| pGEX-4T-1-KD-EphA2R | | CAGGATGCTGACGATGTCAGCGA |
| pHSV1-UL30-C-F | | CGCCTCGAGGTGCGAAAAGACGTTCACCAAGCTG |
| pHSV1-UL30-C-R | | CGCGAATTCCGACGAGTTTCCTCCGCCGTAG |
| **For qPCR analysis** | | |
| qPCR-LANA F | CCTGGAAGTCCCACAGTGTT | |
| qPCR-LANA R | AGACACAGGATGGGATGGAG | |
| qPCR-RTA F | AGACCCGGCGTTTATTAGTACGT | |
| qPCR-RTA R | CAGTAATCACGGCCCCTTGA | |
| qPCR-PAN F | GCCGCTTCTGGTTTTCATTG | |
| qPCR-PAN R | TTGCCAAAAGCGACGCA | |
| qPCR-POV1 F | AGTGCTGTGTTCGCCTTG | |
| qPCR-POV1 R | CACCTCAGAGCCGCTAAG | |
| qPCR-NCOA2 F | GCTGGGAGGACCTGGTAAGA | |
| qPCR-NCOA2 R | ATTTGACTGAATGCCAATCCT | |
| qPCR-PSA F | CACCTGCTCGGGTGATTCTG | |
| qPCR-PSA R | CCACTTCCGGTAATGCACCA | |
| qPCR-UL30-F | AGAGGGACATCCAGGACTTTGT | |
| qPCR-UL30-R | CAGGCGCTTGTTGGTGTAC | |
